# The N-Terminus of CD14 Acts to Bind Apoptotic Cells and Confers Rapid-Tethering Capabilities on Non-Myeloid Cells

**DOI:** 10.1371/journal.pone.0070691

**Published:** 2013-07-30

**Authors:** Leanne Thomas, Anne Bielemeier, Peter A. Lambert, Richard P. Darveau, Lindsay J. Marshall, Andrew Devitt

**Affiliations:** 1 School of Life & Health Sciences & Aston Research Centre for Healthy Ageing, Aston University, Birmingham, United Kingdom; 2 Department of Periodontics, University of Washington, Seattle, Washington, United States of America; National Institute of Biological Sciences, China

## Abstract

Cell death and removal of cell corpses in a timely manner is a key event in both physiological and pathological situations including tissue homeostasis and the resolution of inflammation. Phagocytic clearance of cells dying by apoptosis is a complex sequential process comprising attraction, recognition, tethering, signalling and ultimately phagocytosis and degradation of cell corpses. A wide range of molecules acting as apoptotic cell-associated ligands, phagocyte-associated receptors or soluble bridging molecules have been implicated within this process. The role of myeloid cell CD14 in mediating apoptotic cell interactions with macrophages has long been known though key molecules and residues involved have not been defined. Here we sought to further dissect the function of CD14 in apoptotic cell clearance. A novel panel of THP-1 cell-derived phagocytes was employed to demonstrate that CD14 mediates effective apoptotic cell interactions with macrophages in the absence of detectable TLR4 whilst binding and responsiveness to LPS requires TLR4. Using a targeted series of CD14 point mutants expressed in non-myeloid cells we reveal CD14 residue 11 as key in the binding of apoptotic cells whilst other residues are reported as key for LPS binding. Importantly we note that expression of CD14 in non-myeloid cells confers the ability to bind rapidly to apoptotic cells. Analysis of a panel of epithelial cells reveals that a number naturally express CD14 and that this is competent to mediate apoptotic cell clearance. Taken together these data suggest that CD14 relies on residue 11 for apoptotic cell tethering and it may be an important tethering molecule on so called ‘non-professional’ phagocytes thus contributing to apoptotic cell clearance in a non-myeloid setting. Furthermore these data establish CD14 as a rapid-acting tethering molecule, expressed in monocytes, which may thus confer responsiveness of circulating monocytes to apoptotic cell derived material.

## Introduction

The essential feature of apoptosis *in vivo* is the highly orchestrated clearance of dying cells by phagocytes. This complex multistage process comprises attraction to and recognition, tethering and phagocytosis of cell corpses, and is the net result of the acquisition of neo-antigens (with the most widely characterised example being the exposure of the phospholipid phosphatidylserine [1]) and the loss of inhibitory signals (e.g. CD31 [2] and CD47 [3]) at the dying cell surface. Apoptotic cells (AC) are phagocytosed by local, viable neighbouring cells and it has been suggested that a majority of cell deaths *in vivo* may be cleared by such ‘amateur’ phagocytes. However, when the level of cell death exceeds local corpse-clearance capacity (e.g. in lymphoid follicles [4], acute inflammatory sites [5] or some tumours [6]) professional phagocytes (i.e. macrophages) are recruited by dying cells [7–10] to scavenge persisting dead and dying cells [11]. Most human research in the field has addressed ‘professional’ clearance of AC by macrophages due to the importance in resolution of acute inflammation and during development [12–16]. However AC clearance by non-professional phagocytes (e.g. endothelial/epithelial cells) is well established though our knowledge and understanding of the mechanisms involved is relatively sparse [17–22].

Removal of AC utilises a range of phagocyte receptors that bind, directly or indirectly (via soluble opsonic molecules), to AC and function in a phagocytic synapse (reviewed [6,11,13,23]). Many of these receptors and soluble opsonins are components of the innate immune system (e.g. CD14, complement components, collectins and pentraxins) i.e. are pattern recognition receptors (PRR) - receptors proposed to bind conserved molecular structures on microbes (pathogen-associated molecular patterns, PAMPs e.g. LPS) to activate immune responses [24]. Consequently it has been suggested that AC bear PAMP-like structures named ‘apoptotic cell-associated molecular patterns’ (ACAMPs) that are ligands for PRR (e.g. CD14) mediating AC clearance [25,26]. In support of this, LPS-like structures have recently been revealed on cells undergoing apoptosis [27].

The most striking difference between PRR ligation by PAMPs or ACAMPs lies in the cellular responses. CD14 binds LPS to generate pro-inflammatory responses [28] whilst CD14 promotes AC binding and clearance *in vitro* and *in vivo* in a non-inflammatory manner [29,30]. Thus CD14 ligation with different ligands (PAMP or ACAMP) leads to opposing responses and the molecular basis for this is yet to be defined though a number of models have been proposed [11]. Early mAb studies provide preliminary evidence that LPS and AC may bind to similar regions of CD14 [29]. However AC binding to CD14 remains to be finely mapped and differential ligation of CD14 may underlie the important divergent responses to LPS (pro-inflammatory) or AC (anti-inflammatory). Here we address the key residues of CD14 involved in ligation.

CD14 is an established pro-inflammatory receptor for LPS [31] and other microbial ligands (reviewed [32]) through functional associations with signalling partners such as TLR4/MD2. We hypothesised PAMPs and ACAMPs yield opposing CD14-dependent responses through distinct modes of CD14 ligation thus effecting altered signalling. To this end we have sought to more fully define CD14’s role on monocytes/macrophages and more closely map AC-CD14 binding through a series of targeted point mutants that span the region of CD14 containing LPS binding and signalling sites [33–36] and the region for TLR association [37]. Furthermore we have sought to compare the impact of these mutations on LPS and AC binding and downstream responses.

CD14, a GPI-anchored glycoprotein [38,39] abundant on monocytes and neutrophils, has been used as a marker of monocyte/macrophage lineage, and is considered a myeloid-restricted molecule (reviewed [40]). However we demonstrate CD14 on non-myeloid cells and here characterise CD14 on non-myeloid cells which we report promotes rapid tethering of AC by so called ‘non-professional’ phagocytes thus providing valuable insight to mechanisms by which amateur phagocytes may clear AC.

## Materials and Methods

### Cell lines and culture

All cells were cultured at 37°C in a humidified environment at 5% CO_2_. Mutu I Burkitt’s lymphoma (BL) cells [29,41], U937 (human monocyte line; ATCC) and THP-1 (human myelomonocytic line; LGC Standards-ATCC, Middlesex, UK) were cultured in RPMI 1640 medium. BEAS-2B (normal human bronchial epithelial cells; LGC Standards-ATCC), HEK-293 (human kidney epithelial line; LGC Standards-ATCC), HeLa 229 (human cervical epithelial line; LGC Standards-ATCC), and MCF-7 (human mammary epithelial line; LGC Standards-ATCC) were cultured in DMEM (PAA, Yeovil, UK); Calu-3 (human airway epithelial line; LGC Standards-ATCC) and H400 (human oral epithelial line [42]) were cultured in DMEM/F12 whilst Human Pulmonary fibroblasts isolated from human lung (PromoCell GmbH, Heidelberg, Germany) were cultured in HPFM (PromoCell). All media were supplemented with 2 mM l-glutamine and 10% (v/v) foetal calf serum and 100 IU ml^-1^ penicillin and 100 µg ml^-1^ streptomycin (PAA).

THP-1 cells were stimulated to differentiate towards a macrophage phenotype by 48-72h treatment with 100nM dihydroxyvitamin D3 (VD3; Biomol, Exeter, UK), 250nM phorbol ester (PMA, Sigma, Dorset, UK) or both reagents (VD3/PMA).

### Antibodies

Anti-CD14 monoclonal antibodies 61D3 and 63D3 were obtained as described previously [41] and were produced as tissue culture supernatants whilst MEM18 was purchased (azide-free) from Abcam (Cambridge, UK). All CD14 mAbs were of the isotype IgG1/κ and consequently MOPC21 (Sigma) was used as an isotype matched control. Anti-TLR 4 mAb (HTA125) was supplied unconjugated or as a phycoerythrin-conjugate and matched isotype controls (IgG2a/κ) were supplied by Abcam. Goat anti-mouse secondary reagents used for indirect immunofluorescence studies were used with PE conjugates (Sigma). Sheep anti-mouse-HRP was used for ELISA studies and was supplied by GE Healthcare Life Sciences (Little Chalfont, UK). Sheep anti-human Fc polyclonal antibody was supplied by The Binding Site (Birmingham, UK).

### Recombinant protein expression and analysis

Soluble CD14 expression constructs were generated as previously described [43] to encode CD14 (wild type or point mutant) fused to Fc of human IgG1. These constructs were sub-cloned into a full-length GPI-anchored form by *Hind* III/*Nhe*I excising the 5’ coding region (containing any desired point mutant) and ligating into a similarly cut full length CD14 construct in pcDNA3. The result was a full length CD14 molecule containing the desired point mutants. All mutations were confirmed by sequencing. Soluble CD14-Fc proteins were produced in HEK cells followed *Trans*IT-LT1 (Mirusbio, supplied by Geneflow, Lichfield, UK) mediated cDNA transient transfection as per the manufacturer’s instructions. At 24h post-transfection, medium was replaced with serum-free DMEM and following a further 72 h expressed protein secreted to the supernatant was used directly. For expression of GPI-anchored CD14, *Trans*IT-LT1 mediated transient transfection was again used in the indicated cell lines as per manufacturer’s instructions.

Soluble CD14-Fc constructs were assayed by ELISA. Nunclon Maxisorp 96 well plates (Thermo, Fisher Scientific, Loughborough, UK) were coated with sheep-anti-human at 5µg/ml in carbonate buffer (pH 9.6; Sigma, Dorset, UK). Following washing with PBS(T) (PBS + 0.05% v/v Tween 20), expressed Fc-tagged CD14 proteins were added directly in tissue culture supernatants. The resultant, captured and oriented Fc-fusions were probed with mAbs and bound mAb detected using anti-mouse-HRP and colorimetric SigmaFAST OPD assay (Sigma). In order to control for differing levels of protein production, binding of mAbs was expressed relative to binding of mAb 63D3 whose binding is unaffected by mutation as it binds to the C terminus of CD14 [44,45].

For indirect immuno-fluorescence of cells expressing membrane anchored CD14 constructs (wild type and point mutants), an excess of mAb was incubated with 200,000 cells on ice for 15-30 min, washed in 0.5% (w/v) BSA in PBS and incubated with goat-anti mouse conjugated to PE (1/100 dilution; 100µl volume). Stained cells were analysed either directly or following fixation in 1% w/v formaldehyde in PBS using a Beckman-Coulter Quanta SC. Downstream flow cytometric analyses and presentations were undertaken using FlowJo (Treestar Inc., Oregon, USA).

### Apoptosis induction, detection and photomicroscopy

Mutu I (Burkitt’s lymphoma cells) were exposed to 100 mJ/cm^2^ UV–B irradiation, using a Chromata-vue C71 light box and UVX radiometer (UV–P Inc, USA), and incubated for 16h to allow apoptosis to proceed [10]. For analysis of apoptotic nuclear morphology, cells were fixed in 1% w/v formaldehyde in PBS, stained with 4,6-diamidino-2-phenylindole (DAPI, Sigma, 250ng/ml) and observed using inverted epifluorescence microscopy. For morphological studies, THP-1 cells were seeded to 4 well Lab-Tek II Chamber slides with a coverglass bottom (Thermo, Fisher Scientific) and stimulated to differentiate for 48 hours prior to microscopy. DIC imaging with a 63x (1.4 numerical aperture) Zeiss objective was used for morphology. Nuclei were imaged following acridine orange staining (Sigma, 5µg/ml final concentration). All photomicrography was undertaken using a fully motorised Zeiss Axiovert 200M fluorescence microscope (Carl Zeiss Ltd., Welwyn Garden City, UK) and Hamamatsu Orca camera driven by Volocity (PerkinElmer, Cambridge, UK).

### Apoptotic cell-phagocyte interaction assays

Tethering of AC to phagocytes or interaction (tethering and phagocytosis) of AC with phagocytes was assayed as previously described [46]. Briefly, phagocytes (THP-1-derived or epithelial cells) were seeded to multi-well glass slides [29] and co-cultured with AC (10^6^ per well) for 1hr at 37°C (interaction) or 4°C (tethering) in RPMI containing 0.2% (w/v) bovine serum albumin (Sigma) in the presence or absence of mAbs (1:10 dilution) as appropriate to the experiment. The anti-CD14 mAb 61D3 was used to block CD14-dependent AC interactions with the non-blocking anti-CD14 mAb 63D3 used as a control [29]. Unbound cells were removed by extensive washing and slides fixed in methanol, stained with Jenner/Giemsa (Thermo, Fisher Scientific) and mounted in DPX (Thermo, Fisher Scientific) prior to examination by light microscopy. Binding was undertaken at reduced temperatures, as indicated, with pre-cooled solutions and cell suspensions. In all cases at least 200 macrophages were assessed in each of quadruplicate wells. Data are presented as the percentage of phagocytes binding or interacting with AC. Where transfected HeLa cells were used as phagocytes, cells were reseeded to 4 well glass slides at 24h post-transfection and allowed to stick and spread for 18h prior to use.

### Assays of cell responses to LPS

LPS from *E. coli* O111:B4 (Sigma) was applied to cells (THP-1-derived macrophage cells or epithelial cells (including transfectants) in 24 well plates in the presence of 10% normal human serum (Sigma) as a source of LPS-binding protein. Following indicated time-periods, tissue culture supernatants were harvested and analysed by ELISA for secreted cytokines TNF-α (R&D systems, Abingdon, UK) or IL-8 (Peprotech EC Ltd., London, UK).

In order to specifically assess NFκB transcriptional activity, a luciferase-based NFκB reporter construct (pGL4.32 NFκB reporter; Promega, Southampton, UK) was co-transfected transiently into HeLa cells using *Trans*IT-LT1. Following cell stimulation with LPS or experimental treatments, luciferase activity using One-Glo (Promega) was quantified using an Orion II microplate luminometer (Berthold Detection Systems, Pforzheim, Germany).

### Statistical Analysis

Statistical analysis of results was undertaken using one-way analysis of variance (ANOVA) with a post-test as appropriate to the experiment. All analyses were based on a minimum of three independent experiments and were undertaken using InStat (GraphPad, La Jolla, CA, USA).

## Results

### Expression and function of CD14 on undifferentiated and differentiated THP-1 cells

CD14 expression on myeloid cells (e.g. monocytes, macrophages and neutrophils) is well established though characterisation of function has been largely limited to LPS-induced cytokine responses. We have made significant use of the human monocyte line THP-1 as a source of differentiated myeloid-derived cells expressing CD14. Our initial studies sought to characterise this new panel of macrophage model cells with respect to cell phenotype and function in tethering and mediating responses to AC and LPS with specific relevance to CD14.

**Figure 1:**
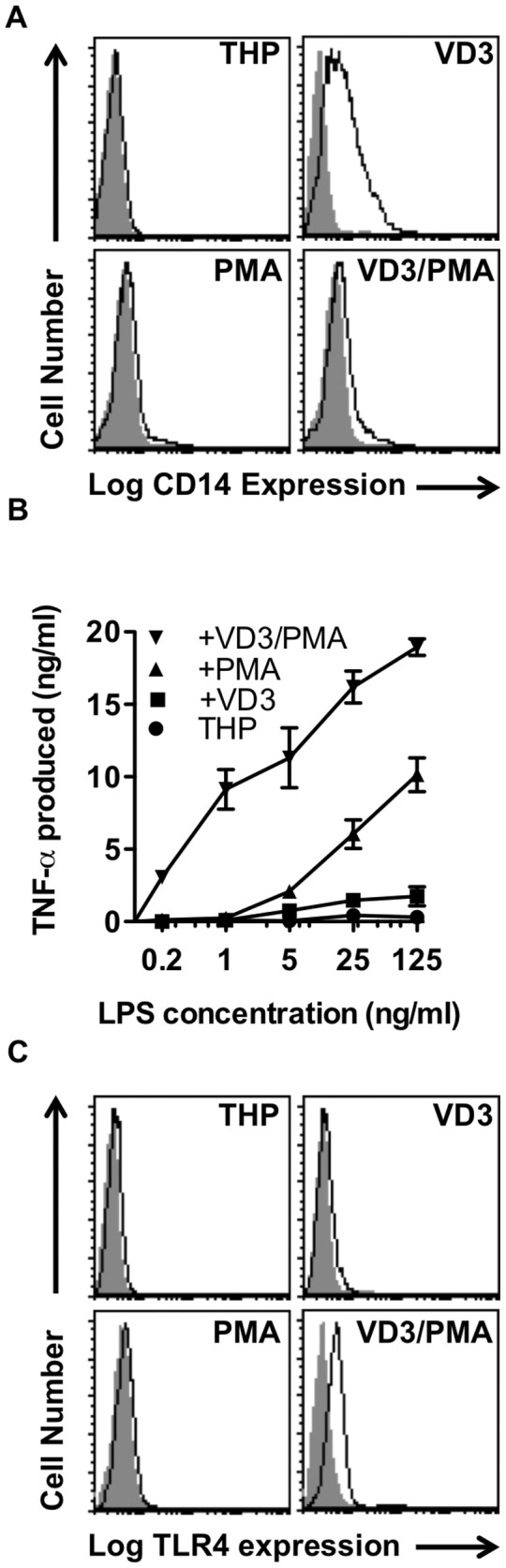
Characterisation of THP-1 macrophage phenotype and LPS responses. THP-1 monocytes (THP-1) cells were stimulated to differentiate in the presence of dihydroxyvitamin D3 (VD3), phorbol ester (PMA) or both (VD3/PMA) for 72 hours prior to analyses. (A) Flow cytometric analysis of cell surface CD14 expression using indirect immunofluorescence with mAb 63D3 detected with anti-mouse-phycoerythrin. Frequency histograms of at least 5000 events are shown for each cell type (open black: CD14; solid grey: IgG1/κ isotype control). Data shown are representative of at least three independent experiments. (B) Production of TNF-α in response to a range of LPS concentrations for 4h in the presence of 10% v/v normal human serum, detected by ELISA (data shown are mean ± SE for 3 independent experiments). (C) Flow cytometric analysis of cell surface TLR-4 using direct immunofluorescence with mAb HTA-125-phycoerythrin (open black: anti-TLR-4; solid grey: IgG2a/κ isotype control). Data shown are representative of at least three independent experiments.

Treatment of THP cells with dihydroxyvitamin D3 (VD3), PMA or both (VD3/PMA) yields three distinct macrophage-like cells when analysed for phenotype and function. Treatment with VD3 alone had no detectable effect on cell volume, granularity or cell division suggesting little stimulation of THP cells from their basal monocyte phenotype whilst treatment with PMA or VD3/PMA had profound effects with increased cell volume (Figure S1A) and reduced cell number after 72h stimulation (Figure S1B). Additionally, only THP-PMA or THP-VD3/PMA showed increased adhesion to plastic (assessed by ease of cell lifting with EDTA treatment: data not shown) with profound morphological changes, suggesting a more advanced, macrophage-like phenotype (Figure S1C). Notably, the different models possessed a range of CD14 expression profiles (Figure 1A) with THP-VD3 cells expressing highest levels of CD14, clearly demonstrating differentiation despite little morphological change. These results revealed an opportunity to assess if CD14 handles AC and LPS in a distinct manner that might underlie divergent cell responses to these ligands. Thus here we describe a powerful range of cell types that are amenable to a range of high-throughput and high-content assays.

To assess LPS sensitivity, TNF-α production in response to a range of LPS concentrations was assayed for each model system and revealed profoundly different responses (Figure 1B) that did not directly correlate with CD14 expression (Figure 1A). Whilst THP-VD3 expressed most CD14 they were not so responsive as THP-VD3/PMA or THP-PMA cells. Further analysis of TLR-4, the well-established LPS-receptor with which CD14 cooperates for the efficient response to LPS, indicates that the greatest LPS-responsiveness correlated with the highest TLR-4 expression (Figure 1C). These data suggest that high CD14 expression is not sufficient for, and high TLR-4 surface expression is not essential for, strong LPS responses. Thus these different model systems provide a valuable opportunity to assess the roles of CD14 and TLR-4 in tethering of and responses to AC.

**Figure 2:**
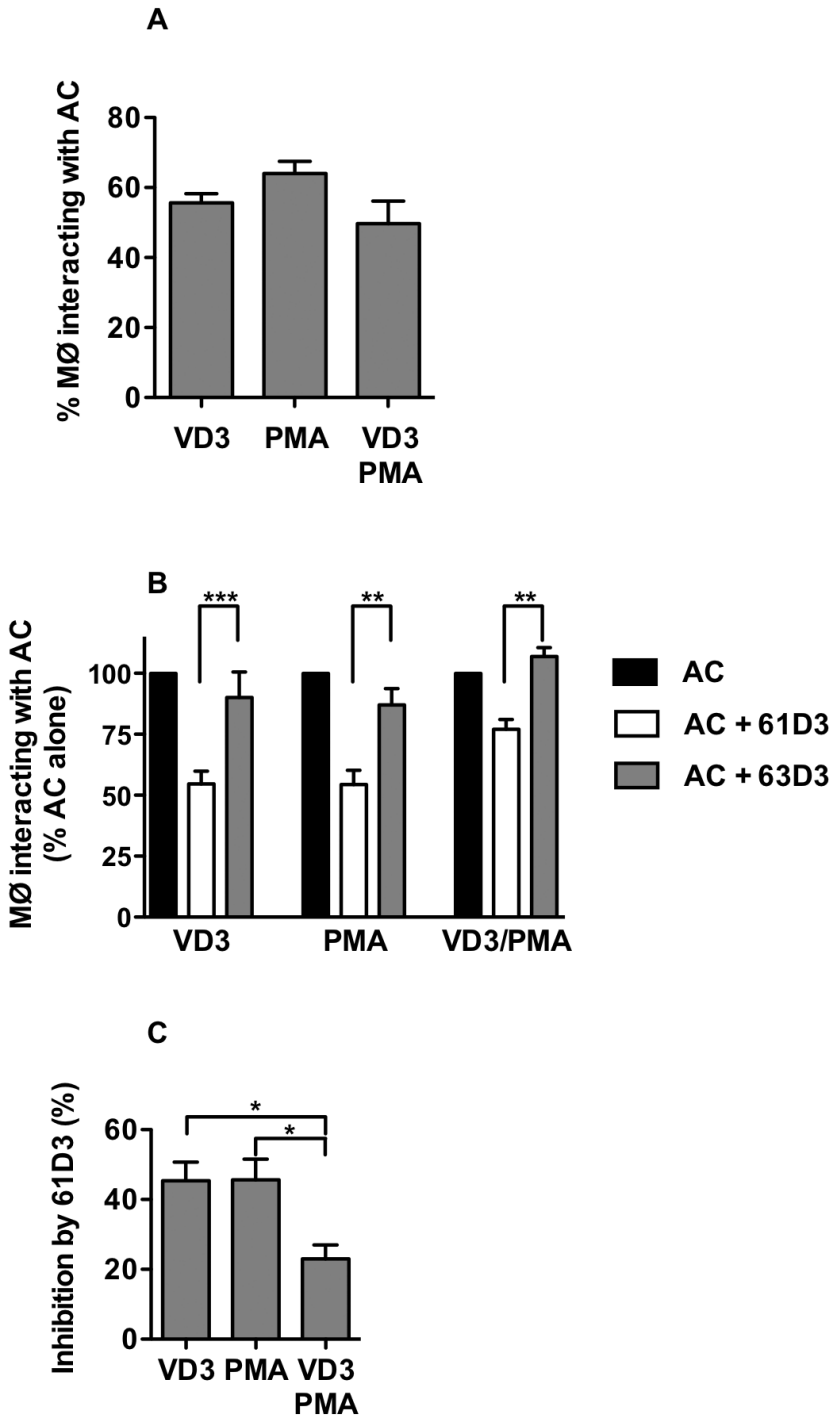
All THP-1 cell-derived macrophages utilise CD14 for interaction with apoptotic cells. UV-induced apoptotic BL cells (>80% apoptotic as assessed by nuclear morphology) were co-cultured with THP-1 phagocytes in the presence of the indicated mAbs (anti-CD14 mAb 61D3, a blocking CD14 mAb, or anti-CD14 mAb 63D3, a non-blocking, isotype matched control). Following co-culture (1h) at 37°C unbound apoptotic cells were removed and the interaction of phagocytes with apoptotic cells assessed by light microscopy of Jenner-Giemsa stained cells. (A) shows the percentage of phagocytes interacting with apoptotic cells. (B) shows the effect of 61D3 and 63D3 on apoptotic cell interaction with phagocytes (% of AC alone). (C) Compares the 61D3 inhibition as a measure of CD14 function for each macrophage type. All data shown are mean ± SE for 7 independent experiments. Statistical analyses used ANOVA with Bonferroni post test. *P<0.05; **P<0.01; ***P<0.001.

To assess the ability of these different macrophage-like cells to interact with AC, differentiated THP-1 cells were co-cultured with AC and the level of phagocyte-AC interaction quantified. Our studies indicate that all three cell types are capable of interacting with AC to a similar degree (Figure 2A) and they did not display the variation noted with LPS responses (Figure 1). We further sought to address CD14’s contribution to AC interaction in each cell systems through the use of the well-established CD14 inhibitory mAb 61D3 and its non-inhibitory counterpart 63D3 [29,41]. In each case CD14 was involved (Figure 2B) in AC-phagocyte interaction with the involvement most profound in THP-VD3 and THP-PMA (Figure 2C), cells that exhibit dramatically different CD14 expression levels (Figure 1A). These data demonstrate CD14 is most active for AC interaction in those cells (VD3 or PMA) where, irrespective of absolute CD14 expression, TLR-4 expression was lowest (i.e. with limited or undetectable TLR-4). This suggests low levels of CD14 are sufficient for AC interaction and TLR-4 is not necessary for CD14-dependent clearance of AC. Indeed, the involvement of CD14 in AC clearance negatively correlates with TLR-4 surface expression.

**Figure 3:**
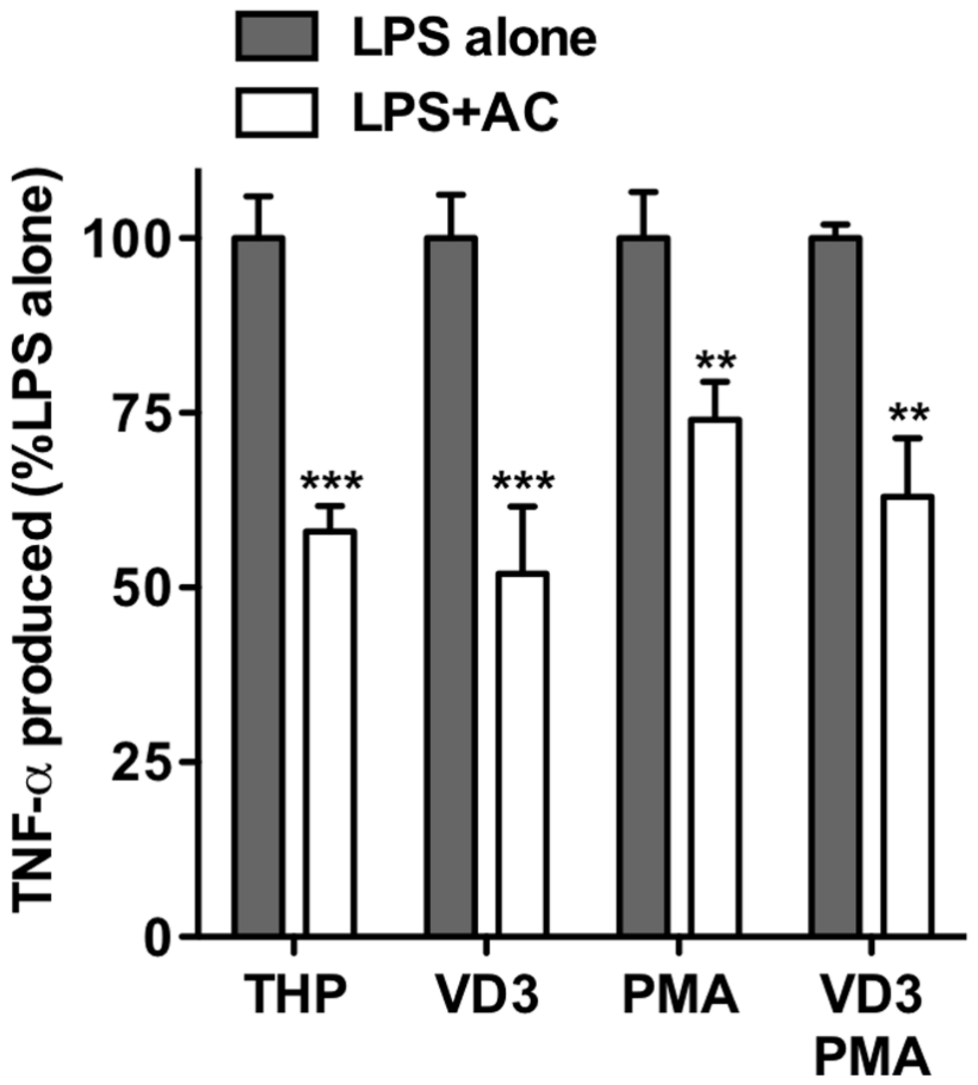
THP-1 macrophage responses to LPS in the presence of apoptotic cells. THP-1 cells or THP-1-derived macrophages (VD3, PMA or VD3/PMA differentiated) were co-cultured with UV-induced apoptotic human B cells for 18h prior to stimulation with LPS. Following 4h stimulation, supernatants were assayed for TNF-α by ELISA. Data presented are TNF-α produced in the presence of LPS and apoptotic cells (LPS+AC) as a percentage of that produced with LPS alone (i.e. no AC added) for each cell type. Data shown are mean ± SE of four independent experiments. Statistical analyses used ANOVA with Bonferroni post-test. **P<0.01; ***P<0.001.

Thus these data may suggest a key difference in the generation of cell responses elicited following CD14 ligation. We further sought to address the ability of each of these THP-1 model systems to elicit an inflammatory response to LPS and for this to be attenuated by AC. In all cases AC reduced the inflammatory response (Figure 3) irrespective of CD14 and TLR-4 expression or LPS-responsiveness.

### Different anti-CD14 mAbs that block AC clearance bind distinct residues in CD14

To closely map CD14 residues involved in AC handling, a series of CD14 point mutants (used previously to analyse the binding of labelled LPS [43]) were used to characterise the interaction with AC. This work was thus designed to address the hypothesis that the divergence of phagocyte responses to CD14 ligation by LPS or AC arises from the key residues involved being different.

**Figure 4:**
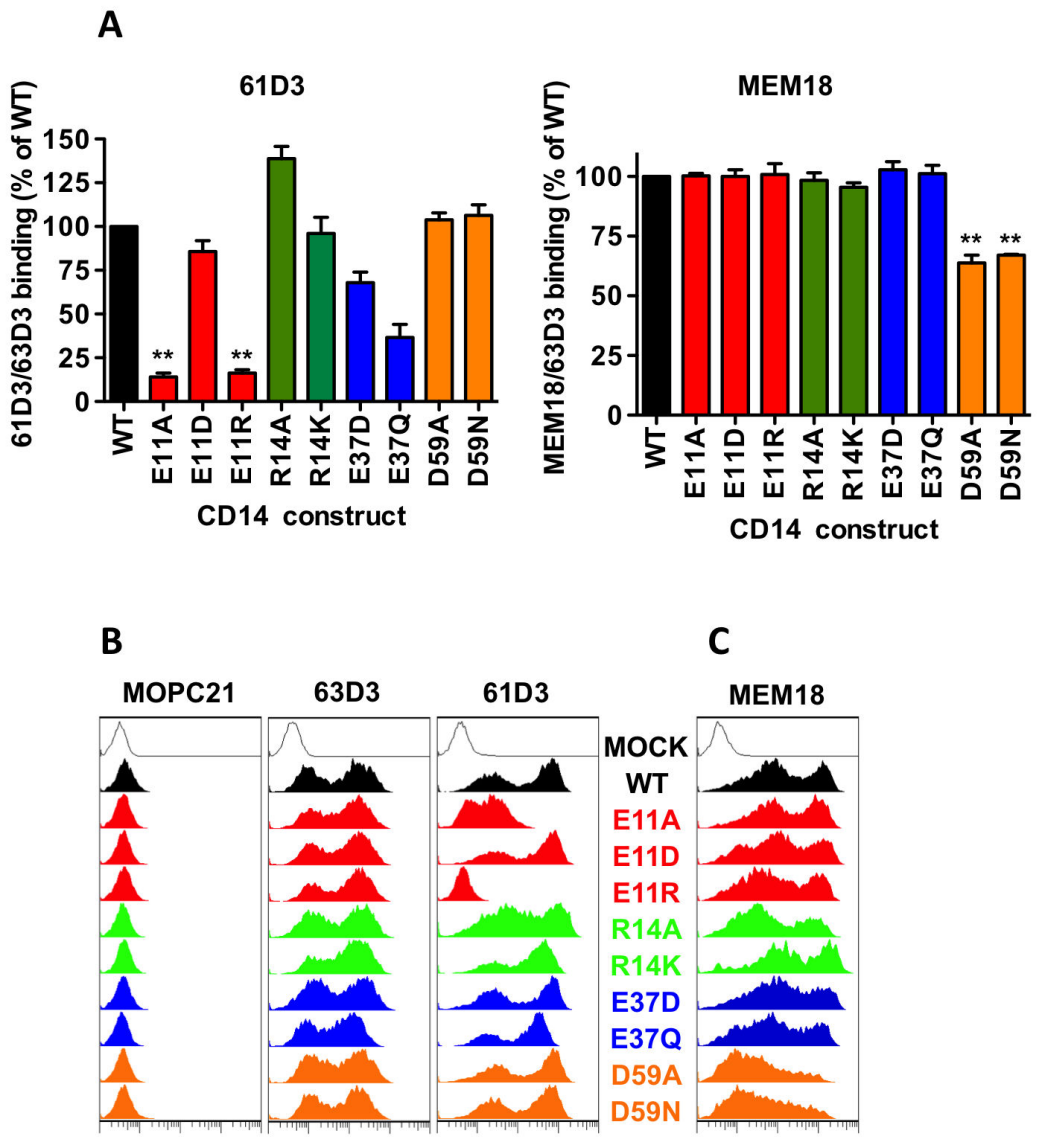
Mapping of key residues within CD14 that are required for binding of mAbs 61D3 and MEM18. Monoclonal Abs 61D3 and MEM18 were tested for reactivity against wild-type CD14 and a panel of point mutants. (A) Anti-human Fc immobilised soluble CD14-Fc fusion proteins were probed by ELISA with 61D3 or MEM18 and mAb binding detected with anti-mouse-HRP prior to developing with OPD substrate and reading OD492nm. mAb is shown as a ratio of 63D3 binding to control for protein expression. Data shown are mean ± SE of three independent experiments and mAb (61D3 or MEM18) binding (relative to 63D3) is expressed as a % of binding to wtCD14Fc. Statistical analysis conducted was ANOVA followed by Dunnett’s post-test (**P<0.01 compared to wtCD14). (B) HeLa cells were transfected with the indicated membrane CD14 (WT or mutant) prior to staining with IgG1/κ isotype control MOPC21, anti-CD14 mAb 63D3, or (C) anti-CD14 mAb MEM18. In each case, mAb binding was detected using goat anti-mouse-PE and flow cytometry. Fluorescence data are shown as histograms with at least 5000 events per plot and are representative of at least three independent experiments.

Previous work shows mAbs 61D3 and MEM18 compete for CD14 binding suggesting epitope similarity [29,47] and both mAbs block AC tethering, suggesting their potential use as surrogate markers of AC binding to CD14. Here we extend those studies to screen mAb binding (by ELISA) to a panel of soluble CD14-Fc (sCD14-Fc) point mutants that span the N-terminal 60 amino acids of CD14 known to be essential for LPS binding and signalling sites. Binding of non-blocking mAb 63D3 was unaffected by any mutation, consistent with it binding CD14’s C-terminus [44,45] and thus confirmed equivalent expression of these mutants (Figure S2A). However, binding of 61D3 (but not MEM18) was significantly reduced by replacement of residue 11 with a neutral or positively-charged residue suggesting a negative charge is required at this site for 61D3 binding (Figure 4A). Additionally mutation of residue 37 showed partial inhibition of 61D3 binding. Consistent with previous work, mutation of residue 59 inhibited binding of MEM18 [33,36] (Figure 4A, right panel) but not 61D3. Thus these two mAbs that both block LPS responses and AC clearance exhibit profoundly different binding patterns to CD14.

To exclude the possibility that these results were soluble CD14-specific, similar mapping studies were undertaken using membrane CD14 (mCD14: WT and mutants) expressed in HeLa cells (chosen for their strong adhesion, ease of transfection and, for later experiments, low basal AC tethering). Binding of mAbs 61D3 and 63D3 to mCD14 mutants was assessed via immunofluorescence and flow cytometry (Figure 4B). Equivalent binding of 63D3 to all mCD14 constructs revealed robust mCD14 expression in a characteristic bi-modal distribution (Figure 4B). Similar mapping was undertaken with MEM18 (Figure 4C). Consistent with our sCD14 mAb mapping, these cell-based assays revealed 61D3 (but not MEM18) binding was profoundly inhibited by residue 11 mutation whilst MEM18 (but not 61D3) binding was inhibited by mutation of residue 59. Regression studies indicate that 61D3-CD14 binding (WT or mutant) was similar for sCD14 and mCD14 (Figure S2C: correlation coefficient r^2^=0.82).

Despite these clear differences in key residues for 61D3 and MEM18 binding previous work [29], repeated here (Figure S3) has indicated that mAbs 61D3 and MEM18 compete for CD14 binding suggesting close proximity of their epitopes and thus residues 11 and 59. This is further supported by the functional observations that both mAbs block AC binding.

### CD14 residue 11 is essential for apoptotic cell binding

**Figure 5:**
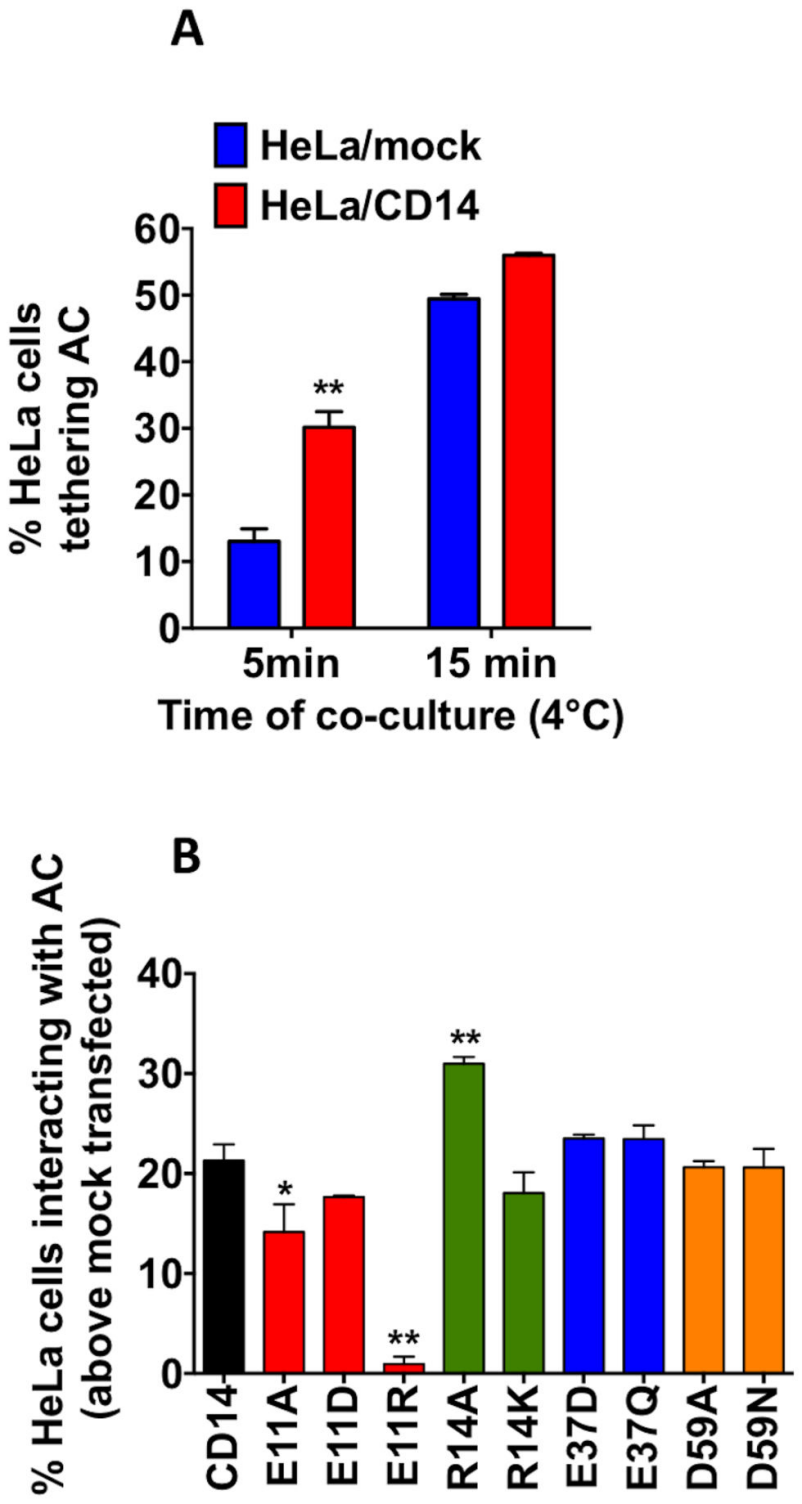
Residue 11 of CD14 is essential for binding to apoptotic cells. HeLa cells were used as surrogate phagocytes and co-cultured with apoptotic human B cells. Following co-culture at 4°C unbound apoptotic cells were removed and the interaction of phagocytes with apoptotic cells assessed by light microscopy of Jenner-Giemsa stained cells. (A) HeLa cells (mock or CD14WT transfected) co-cultured with UV-induced apoptotic human B cells (>80% apoptotic as assessed by nuclear morphology) for 5 or 15 min were used to assess the optimal period to reveal the role of CD14 to promote apoptotic cell binding. Data shown are mean ± SE for three independent experiments. Statistical analyses used ANOVA with Tukey post-test. **P<0.01. (B) HeLa cells transfected with CD14WT or point mutants were used as surrogate phagocytes and co-cultured with apoptotic human B cells for 5 min. Following co-culture at 4°C unbound apoptotic cells were removed and the interaction of phagocytes with apoptotic cells assessed by light microscopy of Jenner-Giemsa stained cells. Data are shown as the percentage of HeLa cells interacting with apoptotic cells (mean ± SE above the binding to HeLa mock) for three independent experiments. Statistical analyses used ANOVA with Dunnett’s post-test. *P<0.05; **P<0.01.

Extending our CD14 mutant mapping studies from the use of mAbs as surrogate markers of AC binding, we sought to map AC binding to our panel of CD14 constructs. Use of soluble CD14 mutants was not possible due to the inherent variability noted between binding experiments. Hence, mCD14 constructs were screened using HeLa cells transiently transfected with mCD14 and an assay was developed that made use of low-temperature to prevent phagocytosis whilst permitting tethering to proceed. To assess CD14 function in this system, we established an assay system that revealed improved AC tethering following wt-mCD14 expression (akin to previous non-human systems [29]). Following extensive work an assay was optimised that assessed AC tethering to phagocytes in a 5 minute co-culture at 4°C. This assay demonstrated CD14’s ability to significantly improve AC tethering capacity of non-professional phagocytes by 100% (Figure 5A). Extended periods (15 minutes+) showed higher levels of AC tethering that masked CD14’s function (Figure 5A), presumably as other redundant receptors came into play. This suggests CD14 is a key molecule in the rapid, earliest binding events of professional phagocytes and may have important implications for the function of circulating monocytes responding to material released from AC (e.g. from sites of apoptosis such as tumours and atherosclerotic plaques).

Using this developed assay system, mCD14 constructs (WT and mutant) were transiently expressed in HeLa cells and CD14 expression assessed using mAb 63D3, known to bind away from the mutated regions [44,45]. Flow cytometric analysis demonstrated successful, equivalent expression of all mCD14 mutants (Figure S2B shows representative expression data). The characteristic bi-modal expression distribution (e.g. Figure 4B Figure S2B) was noted in all cases and also with control transfection with a reporter GFP plasmid suggesting this is a feature of HeLa cell transfection rather than a CD14-specific effect.

Results from AC tethering to these HeLa/mCD14 cells are shown (Figure 5B). We demonstrate that glutamic acid at residue 11 (key for 61D3 binding) was also a key residue for AC tethering. Importantly this mutation does not destroy the overall confirmation of CD14 as all other mAbs tested bind well. Also residue 59 (important for MEM18 binding) was mutated without altering the ability of CD14 to mediate AC tethering. Thus we believe 61D3 to be a good surrogate marker of AC binding and for residue 11 to be key for tethering of AC by CD14.

### CD14-mediated responses

**Figure 6:**
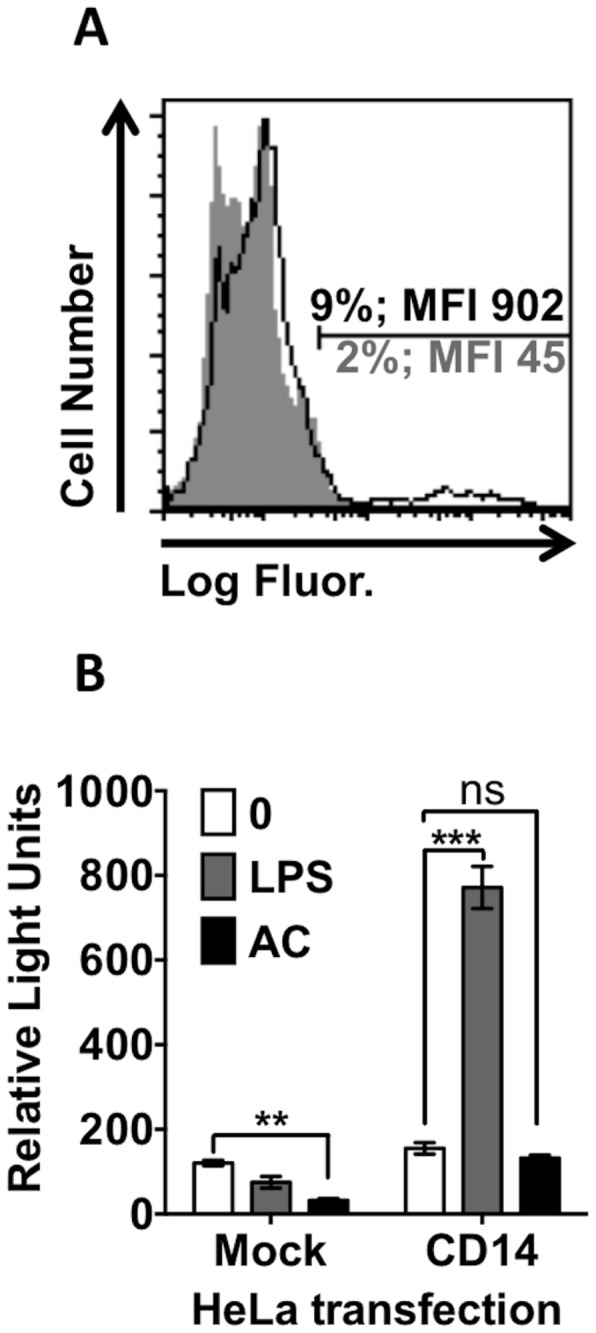
LPS but not apoptotic cells activates NFκB inflammatory signalling. HeLa cells were transfected with both the luciferase NFκB reporter plasmid and a CD14WT expression plasmid or ICAM-3 expression plasmid as a control using *Trans*IT LT-1. Expression was allowed to proceed for 24 hours prior to further analyses. (A) CD14 expression was assessed using indirect immunofluorescence with mAb 63D3 (open black) detected using goat anti-mouse PE, compared to isotype control stained cells (solid grey). (B) Cells were treated with either 100µg/ml of LPS or apoptotic human B cells for 5h prior to assessing NFκB-mediated transcriptional activity with One-Glo Luciferase assay system. Apoptotic cells were in excess of 80% apoptotic by nuclear morphology. Relative light units were quantified using a microplate luminometer. The data shown is mean ± SE of three independent experiments. Statistical analyses used ANOVA with Tukey post-test. **P<0.001; ***P<0.001; ns = not significant.

Divergent responses emanating from CD14 ligation may be due to alternative ligation of CD14 by different ligands. To this point, our data support the notion that AC and LPS ligate CD14 using at least some similar residues. Consequently, one might predict pro-inflammatory signalling to occur following LPS or AC binding to CD14 and this signalling be inhibited downstream. To test this, we used an NFκB reporter in HeLa/mCD14 cells to report pro-inflammatory transcriptional activation. Initial work titrated LPS on HeLa/CD14 cells versus HeLa/mock transfected cells to ascertain the minimum LPS concentration that would induce a significant CD14-dependent response to LPS (Figure S4). This LPS concentration (100µg/ml) was thus used for future experiments. We note strong LPS-induced transcriptional activation when CD14 is expressed, confirming inflammatory signalling (Figure 6B). However, AC fail to induce detectable NFκB-dependent transcription in HeLa/CD14 cells above background, suggesting ligation of CD14 by AC does not activate NFκB responses. Of interest though is the observation that AC reduce the baseline response in HeLa/mock cells, suggesting AC are capable of modulating NFκB responses by a CD14-independent mechanism.

### Non-myeloid CD14 mediates apoptotic cell clearance

**Figure 7:**
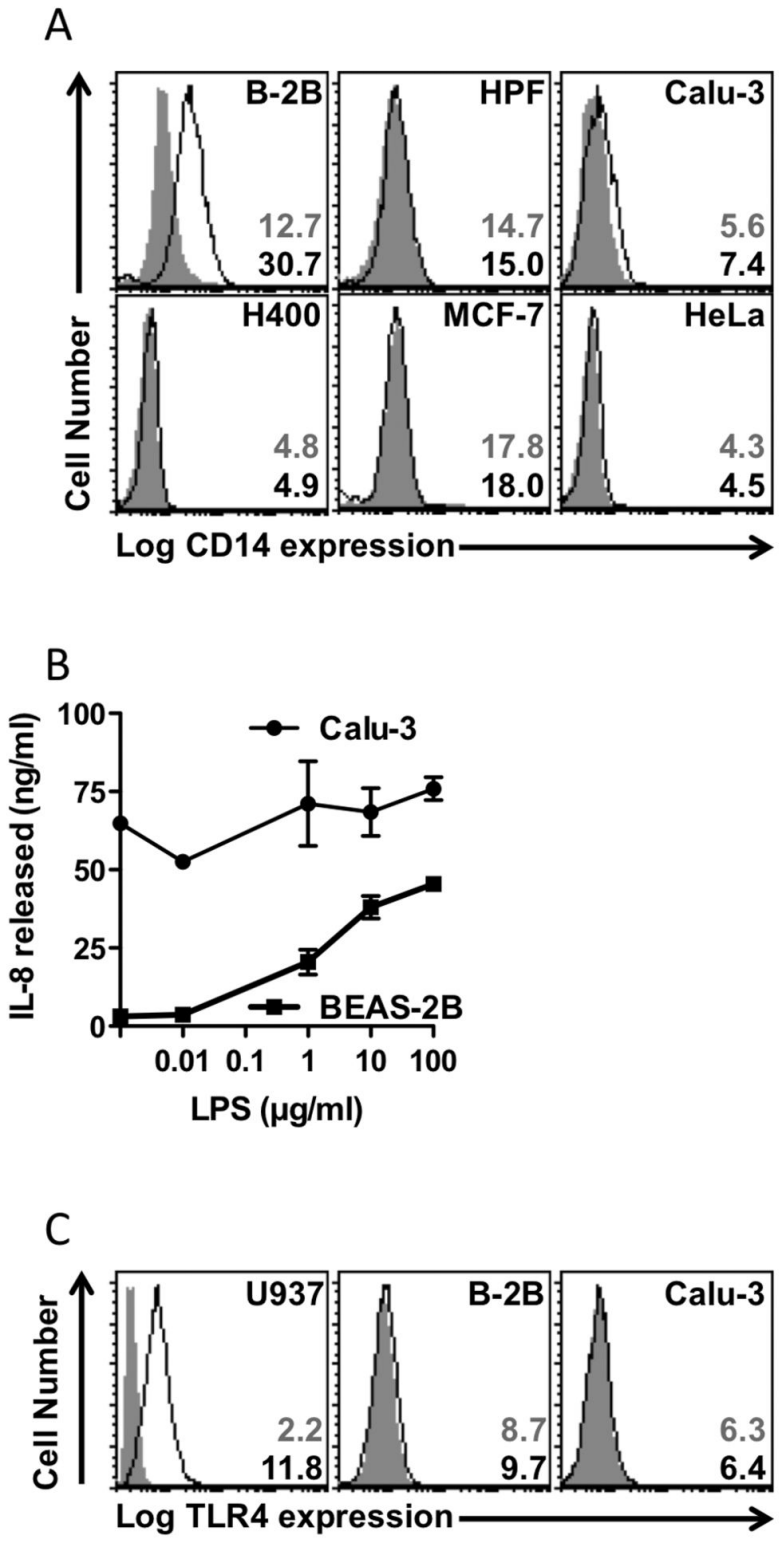
CD14 is expressed in non-myeloid cells but is not sufficient for inducible LPS responses. (A) Flow cytometric analysis of cell surface CD14 expression on a panel of non-myeloid cells. Cells were assessed for cell surface CD14 through the use indirect immunofluorescence with anti-CD14 mAb 63D3 (or MOPC21 isotype control) and detected with goat anti-mouse-phycoerythrin. Frequency histograms of at least 5000 events are shown for each cell type (red: CD14; grey: IgG1/κ isotype control). Numerical values shown are the mean fluorescence intensity for anti-CD14 (open black) or isotype control (solid grey) stained cells. (B) IL-8 production by CD14-expressing BEAS-2B and Calu-3 cells following LPS treatment at the indicated concentrations for 24 hours. ELISA of supernatant IL-8 was undertaken and results shown are mean ± SE of three independent experiments. (C) Flow cytometric analysis of cell surface TLR4 expression on BEAS-2B and Calu-3 cells. Cells were assessed for cell surface TLR4 by direct immunofluorescence with PE-conjugated anti-TLR4 mAb HTA125 (or an isotype control). Frequency histograms of at least 5000 events are shown for each cell type (open black: TLR4-PE; solid grey: IgG2a/κ isotype control-PE). Values shown are the mean fluorescence intensity for anti-TLR4 (black) or isotype control (grey) stained cells.

Our data in HeLa cells suggest strongly that CD14 is a rapid-acting tethering receptor for AC. Whilst screening a panel of epithelial cells as candidates for our CD14 over-expression studies (above) we noted CD14 expression on two pulmonary epithelial cells, BEAS-2B and Calu-3, but not on other epithelial cells or pulmonary fibroblasts tested (Figure 7A) providing a valuable opportunity to assess the function of natural expressed CD14 on non-myeloid, ‘amateur’ phagocytes. Whilst mCD14 is often considered to be myeloid-restricted, a number of studies challenge this (reviewed [40]). In our studies, BEAS-2B expressed robust levels of mCD14 consistent with the cell’s ability to respond to LPS in a dose-dependent manner for the production of IL-8. Calu-3 cells expressed much lower levels of CD14 and were less LPS-responsiveness. Despite expression of CD14 on these cells they were relatively weakly LPS-responsive (Figure 7B in comparison to myeloid cells Figure 1) and this is likely due to the low/negative levels of detectable TLR-4 at the cell surface (Figure 7C).

**Figure 8:**
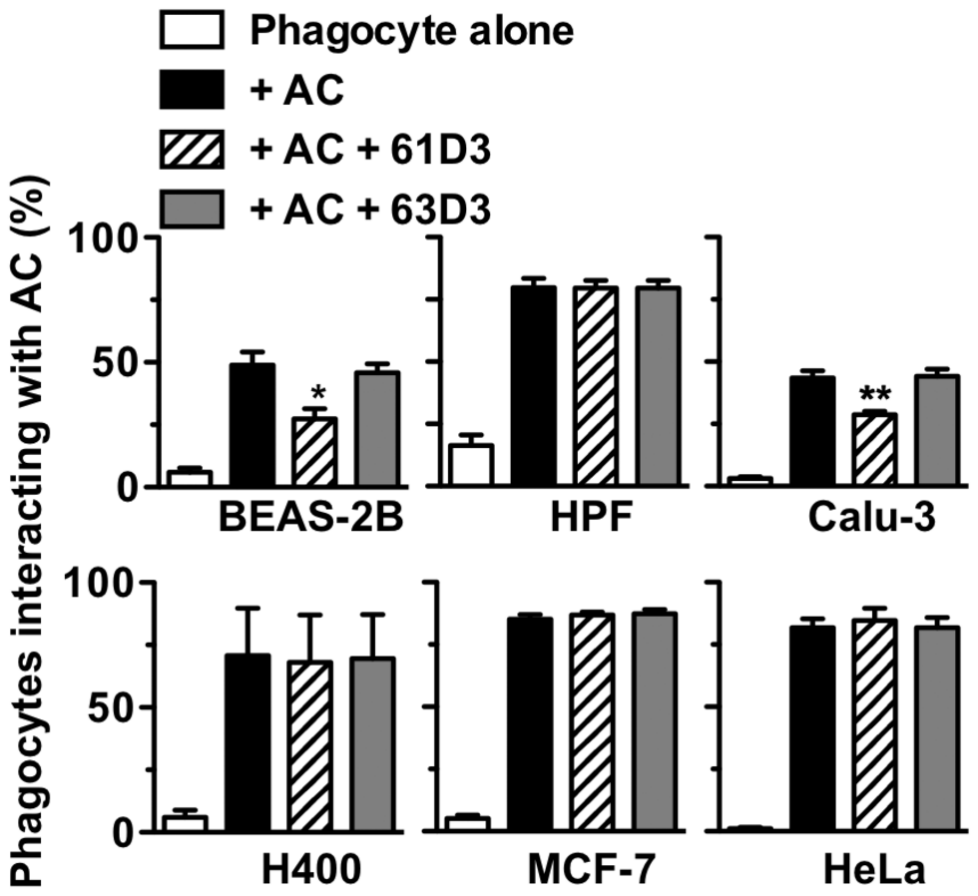
Extramyeloid CD14 is functional to mediate interaction with apoptotic cells. Non-myeloid cells (epithelial cells: BEAS-2B, Calu-3, H400, MCF-7 HeLa or human pulmonary fibroblasts, HPF) were seeded to glass slides and co-cultured with apoptotic human B cells in the absence or presence of anti-CD14 mAbs 61D3 or 63D3. Following co-culture for 1h at 37°C, unbound apoptotic cells were removed by washing and the percentage of non-myeloid phagocytes interacting with apoptotic cells was assessed by light microscopy of Jenner-Giemsa stained cells. Results shown are mean ± SE of three independent experiments. Statistical analyses used ANOVA with Tukey post-test. *P<0.05; **P<0.01.

We further sought to assess, for the first time, the ability of epithelial cell-expressed CD14 to mediate interaction with AC using mAb 61D3 as a tool to block CD14’s AC tethering function. These studies reveal that Beas-2B and Calu-3 both interact with AC in a CD14-dependent manner whilst other cells tested (with no detectable mCD14) did not (Figure 8). The extent of CD14 involvement is significant in both BEAS-2B and Calu-3, even when CD14 expression levels are relatively low, consistent with CD14 playing an important role for tethering AC.

Taken together these data may suggest CD14 on pulmonary epithelial cells primarily promotes AC clearance within the lungs and that poor TLR-4 expression is a safe-guard against chronic stimulation from the lung microbiome [48]. The presence of CD14 on pulmonary epithelial cells may thus promote immobilisation of AC and debris until efficient phagocytosis can occur. Furthermore this may be critical in influencing airways inflammation [42]. Further work is required to establish fully the significance of these CD14 observations.

## Discussion

Whilst previous studies suggest CD14 mediates both LPS-induced inflammation and AC tethering via closely associated regions, the precise residues involved in binding of PAMPs/ACAMPs have not been resolved despite the potential importance in generating opposed cell responses. Herein we characterise CD14’s role in AC tethering and cellular responses through the use of a series of CD14-expressing myeloid and non-myeloid model systems.

We report four different myeloid cell systems (THP-1 cells and three macrophage derivatives with differing levels of CD14 and TLR4) and show, for the first time, markedly different macrophage responses to LPS though similar AC clearance capacity. Whilst CD14 levels do not correlate with the cells’ ability to clear AC (Figures 1 and 2), the most LPS-responsive cells (THP-VD3/PMA) utilise CD14 the least to tether AC (Figure 2C). Furthermore, those cells (THP-VD3; THP-PMA) with lowest TLR-4 expression (irrespective of CD14 levels) used CD14 to the greatest degree in tethering AC (Figure 2C). Taken together these data suggest that TLR-4 expression negatively correlates with CD14’s ability to tether AC, possibly through competition for CD14 binding. This may help explain why TLR-4^-/-^ macrophages take up AC more efficiently than TLR4^+/+^ macrophages [49]. Previous studies have addressed TLR involvement in AC removal as PRRs are proposed to bind ACAMPs - ligands that have recently been revealed on the surface of AC [27]. However TLR4 is involved in phagosome maturation rather than AC-tethering [49]. TLRs have also been implicated in responses to necrotic cells [50] and thus AC may exclude TLR4-MD2 from the CD14 signalling complex within lipid rafts [51], a possible mechanism by which inflammatory signalling from CD14 ligation may be reduced by AC. CD14 interacts with TLR4/MD2 in the presence of LPS, which is sandwiched between CD14 and TLR4/MD2, to effect inflammatory signalling [52]. Thus tethering of AC to CD14 residues required for LPS binding/signalling may outcompete LPS and prevent TLR4 involvement [53].

A further explanation of how LPS and AC may ligate CD14 yet yield opposing responses lies in the precise residues ligated. Here we show two mAbs (61D3 and MEM18), which individually block both LPS responses and AC binding, bind different key residues in CD14 with 61D3 reliant upon residue E11 and MEM18 on residue D59. Given the similar functional effects of these mAbs this may be surprising. However, assuming good structural homology between mouse and human CD14, the residues are topologically close when mapped onto the predicted 3D crystal structure [54]. Within this crystal structure, four previously identified regions essential for LPS binding/signalling [33,34,36,43,55] have been identified and E11 and D59 lie within these regions and are closely-associated with the rim and wall of the LPS-binding pocket of CD14 respectively [54].

Our analyses of mCD14 mutants in HeLa cells indicate that AC tethering, in a novel and robust binding assay, is also dependent on residue E11. Thus this work defines mAb 61D3 as a good surrogate marker for AC tethering by CD14. Our studies suggest that a negative charge is essential at residue 11 for efficient AC binding. Notably, binding of mAb 61D3 to CD14 is more sensitive to mutation in residue 11 than is AC binding. Replacement of an acidic residue with either a basic or neutral residue has profound effects on 61D3 binding. AC binding is much less affected by a neutral substitution at this site. This likely reflects the differences between mAbs and AC as ligands, where AC have many other points if interaction with HeLa cells whilst mAbs bind a single epitope on CD14. This work identifies for the first time the crucial residue in CD14 that is essential for CD14’s function to tether AC.

The precise residues involved in LPS binding are difficult to define and mAb studies have been widely used, with MEM18 reported to compete with LPS for CD14 binding [33] though single residue mutation studies do not reveal residue D59 as essential for binding of *E. coli* LPS [43] with residues 37-44 implicated [36,55]. Notably region 1 of CD14, which includes residue 11 that we show is essential for CD14 tethering of AC, is not essential for LPS binding but is necessary for CD14-dependent activation [34,56]. More detailed mutation studies, incorporating single residue substitution mutations, also demonstrate that residue 11 mutation does not affect binding of *E. coli* LPS [43]. Thus it is possible that AC binding to residue 11 may inhibit LPS signalling and, via competition, also inhibit LPS binding to other essential residues so as to reduce the potential for LPS pro-inflammatory signalling.

Given our observation that a key signalling residue in CD14 is essential for AC tethering, it was possible that AC-CD14 binding was agonistic (i.e. pro-inflammatory). However we demonstrate AC ligation does not stimulate NFκB pro-inflammatory signalling. Thus if CD14 signalling, following AC ligation, is activated, it is inhibited upstream of NFκB. Taking all our data together, it seems most likely that AC prevent CD14 inflammatory signalling by modification of the signalling complex at the cell surface, a possibility that requires further study.

Of relevance to this discussion, is the identity of ligands for CD14 that reside on AC. To date they have not been formally identified though it has been suggested that apoptotic cell-associated ICAM-3 may mediate AC removal through the CD14 pathway [57]. Whilst this is an attractive possibility, there is no evidence to indicate that CD14 and ICAM-3 directly interact. Irrespective of this, CD14 is known to mediate removal of apoptotic cells that do not express ICAM-3, suggesting that other ligands likely exist [47]. AC-associated ligands for PRR (e.g. CD14) have been suggested to share structural similarities with PAMPs and thus be recognisable [25] [26]. Recently a novel strategy to identify ACAMPs has been used successfully, through the use of anti-LPS mAbs. However, whilst this epitope colocalised with annexin V- and C1q-binding sires on AC, it did not appear to interact preferentially with CD14 [27].

Finally whilst establishing our novel human AC tethering assay, we identified airways epithelial cells that naturally express CD14. This extra-myeloid CD14 expression has been the subject of debate but has been reported on primary epithelium from airways and urogenital tract (reviewed [40]). Early studies have shown sCD14 and transfected mCD14 support LPS responses of non-myeloid cells (reviewed [32]). Here we demonstrate that CD14 expressed in HeLa cells significantly improve their ability to tether apoptotic cells in a rapid manner, suggesting that CD14 functions apically in the tethering process. This perhaps accounts for the profound phenotype noted in CD14^-/-^ mice where persisting AC are noted in multiple tissues [46]. Furthermore, we demonstrate, for the first time, that naturally occurring extra-myeloid CD14 is functional for AC tethering. These data suggest that CD14’s primary role in non-myeloid airway cells may be to immobilise AC rapidly and, should the dead cell burden not be too great, to also remove cell corpses. In support of this hypothesis, increases in shed, soluble CD14 (e.g. by human neutrophil elastase, which reduces clearance of AC [58]) is associated with heightened airways inflammatory responses and an increased burden of AC (e.g. in smoking-related emphysema [59]; following allergen challenge of asthmatics [60]). Additionally, reduced AC clearance is linked to lung disease in humans and, in rodent models, induction of apoptosis in the airways leads to pathological changes typical of emphysema (reviewed [61]). Interestingly the relatively poor inflammatory responses of these cells to LPS may be functionally important within the airways. The lungs do not constitute a sterile environment and poor CD14 responses to LPS prevent chronic inflammation in response to the LPS load of the lung microbiome [48]. Furthermore a recent report highlights apoptotic cell clearance by bronchial epithelial cells as a critical influence on airway inflammation [42]. Thus the rapid tethering of apoptotic cells mediated by non-myeloid CD14 may be an important event in the control of pulmonary inflammation.

## Supporting Information

Figure S1 Morphological characterisation of THP cells and their differentiated counterparts. (A) THP-1 monocytes (THP-1) cells were stimulated to differentiate in the presence of dihydroxyvitamin D3 (VD3), phorbol ester (PMA) or both (VD3/PMA) for 48 hours. Resultant cells were detached into 5mM EDTA in PBS by incubation at 37°C for 15 min prior to flow cytometric analysis of cell volume. Data shown are representative of the electronic volume frequency histograms for the resultant cell populations. (B) THP-1 cells were seeded at a density of 5x10^5^ cells per well prior to mock treatment (THP-1) or treatment with 100nM dihydroxyvitamin D3 (VD3), 250nM phorbol ester (PMA) or VD3/PMA. Following 72 hours, cell numbers were assessed using the cell count function of the Quanta SC flow cytometer. Data shown are the mean ± SE of cell counts from three independent experiments. (C) THP-1 monocytes (THP-1) cells were stimulated to differentiate in the presence of dihydroxyvitamin D3 (VD3), phorbol ester (PMA) or both (VD3/PMA) for 48 hours in 4 well chamber slides. Cell nuclei were stained with acridine orange. Representative DIC morphology images overlaid with fluorescence nuclear morphology images of THP-1 cells or the resultant differentiated cell are shown. Multinucleate cells, suggestive of cell fusion, are shown (arrows). Scale bar = 16µm.
(TIF)

Figure S2 Characterisation of HeLa cells transfected with membrane associated CD14 constructs (WT and point mutant). (A) Monoclonal Ab 63D3 was tested for reactivity against wild-type CD14 and a panel of point mutants. Anti-human Fc immobilised soluble CD14-Fc fusion proteins were probed by ELISA with mAb 63D3 and binding detected with anti-mouse-HRP prior to developing with OPD substrate and reading OD492nm. Data shown are mean ± SE of three independent experiments. Statistical analyses indicate no significant difference in response to any of the CD14 constructs (ANOVA with Dunnett’s post-test). (B) HeLa cells were transfected with pcDNA3/GFP. The fluorescence frequency histogram shown reveals the representative bi-modal expression pattern noted in all our HeLa cell studies. (C) Regression analysis of 61D3 mapping studies on soluble CD14 constructs (WT and point mutants) and HeLa cell membrane expressed constructs. Binding of 61D3 to sCD14 is plotted against the mean fluorescence intensity of 61D3 stained HeLa transfectants (all data from Figure 4). This analysis reveals a strong correlation between 61D3 mapping on soluble and membrane CD14 with a correlation coefficient (r) = 0.905.
(TIF)

Figure S3 Monoclonal Ab MEM18 competes with 61D3 for binding to CD14. Anti-human Fc immobilised soluble WT CD14-Fc fusion protein was probed by ELISA with mAb 61D3-biotin and binding of the biotinylated mAb detected with streptavidin-HRP prior to developing with OPD substrate and reading OD492nm. The ability of unlabelled 61D3 (red bar) or unlabelled MEM18 (blue bars, used at indicated concentrations) to block binding of biotinylated 61D3 was assessed. Data shown are mean ± SE of three independent experiments. Statistical analyses used ANOVA with Dunnett’s post-test to detect significant of differences compared to 61D3-biotin alone (black bar).
(TIF)

Figure S4 Assessment of LPS required to activate NFκB inflammatory signalling. HeLa cells were transfected with both the luciferase NFκB reporter plasmid and a CD14WT expression plasmid or ICAM-3 expression plasmid as a control using *Trans*IT LT-1. Expression was allowed to proceed for 24 hours prior to further analyses. Cells were treated with the indicated concentrations of LPS for 5h prior to assessing NFκB-mediated transcriptional activity with One-Glo Luciferase assay system. Relative light units were quantified using a microplate luminometer. The data shown is mean ± SE of three independent experiments. Statistical analyses used ANOVA with Tukey post-test. *P<0.05.
(TIF)

## References

[1] Fadok VA, Voelker DR, Campbell PA, Cohen JJ, Bratton DL et al. (1992) Exposure of phosphatidylserine on the surface of apoptotic lymphocytes triggers specific recognition and removal by macrophages. J Immunol 148: 2207-2216. PubMed: 1545126. 1545126

[2] Brown S, Heinisch I, Ross E, Shaw K, Buckley CD et al. (2002) Apoptosis disables CD31-mediated cell detachment from phagocytes promoting binding and engulfment. Nature 418: 200-203. doi:10.1038/nature00811. PubMed: 12110892. 12110892 10.1038/nature00811

[3] Gardai SJ, McPhillips KA, Frasch SC, Janssen WJ, Starefeldt A et al. (2005) Cell-surface calreticulin initiates clearance of viable or apoptotic cells through trans-activation of LRP on the phagocyte. Cell 123: 321-334. doi:10.1016/j.cell.2005.08.032. PubMed: 16239148. 16239148 10.1016/j.cell.2005.08.032

[4] Hanayama R, Tanaka M, Miyasaka K, Aozasa K, Koike M et al. (2004) Autoimmune disease and impaired uptake of apoptotic cells in MFG-E8-deficient mice. Science 304: 1147-1150. doi:10.1126/science.1094359. PubMed: 15155946. 15155946 10.1126/science.1094359

[5] Savill JS, Wyllie AH, Henson JE, Walport MJ, Henson PM et al. (1989) Macrophage phagocytosis of aging neutrophils in inflammation. Programmed cell death in the neutrophil leads to its recognition by macrophages. J Clin Invest 83: 865-875. doi:10.1172/JCI113970. PubMed: 2921324. 2921324 10.1172/JCI113970 PMC303760

[6] Gregory CD, Pound JD (2010) Microenvironmental influences of apoptosis in vivo and in vitro. Apoptosis 15: 1029-1049. doi:10.1007/s10495-010-0485-9. PubMed: 20237956. 20237956 10.1007/s10495-010-0485-9

[7] Segundo C, Medina F, Rodríguez C, Martínez-Palencia R, Leyva-Cobián F et al. (1999) Surface molecule loss and bleb formation by human germinal center B cells undergoing apoptosis: role of apoptotic blebs in monocyte chemotaxis. Blood 94: 1012-1020. PubMed: 10419893. 10419893

[8] Truman LA, Ford CA, Pasikowska M, Pound JD, Wilkinson SJ et al. (2008) CX3CL1/fractalkine is released from apoptotic lymphocytes to stimulate macrophage chemotaxis. Blood 112: 5026-5036. doi:10.1182/blood-2008-06-162404. PubMed: 18799722. 18799722 10.1182/blood-2008-06-162404

[9] Peter C, Wesselborg S, Herrmann M, Lauber K (2010) Dangerous attraction: phagocyte recruitment and danger signals of apoptotic and necrotic cells. Apoptosis 15: 1007-1028. doi:10.1007/s10495-010-0472-1. PubMed: 20157780. 20157780 10.1007/s10495-010-0472-1

[10] Torr EE, Gardner DH, Thomas L, Goodall DM, Bielemeier A et al. (2011) Apoptotic cell-derived ICAM-3 promotes both macrophage chemoattraction to and tethering of apoptotic cells. Cell Death Differ, 19: 671–9. PubMed: 22117198. 22117198 10.1038/cdd.2011.167 PMC3307987

[11] Gregory CD, Devitt A (2004) The macrophage and the apoptotic cell: an innate immune interaction viewed simplistically? Immunology 113: 1-14. doi:10.1111/j.1365-2567.2004.01959.x. PubMed: 15312130. 10.1111/j.1365-2567.2004.01959.x PMC1782541 15312130

[12] Fadok VA, Bratton DL, Guthrie L, Henson PM (2001) Differential effects of apoptotic versus lysed cells on macrophage production of cytokines: role of proteases. J Immunol 166: 6847-6854. PubMed: 11359844. 11359844 10.4049/jimmunol.166.11.6847

[13] Savill J, Dransfield I, Gregory C, Haslett C (2002) A blast from the past: clearance of apoptotic cells regulates immune responses. Nat Rev Immunol 2: 965-975. doi:10.1038/nri957. PubMed: 12461569. 12461569 10.1038/nri957

[14] Lauber K, Blumenthal SG, Waibel M, Wesselborg S (2004) Clearance of apoptotic cells: getting rid of the corpses. Mol Cell 14: 277-287. doi:10.1016/S1097-2765(04)00237-0. PubMed: 15125832. 15125832 10.1016/s1097-2765(04)00237-0

[15] Elliott MR, Ravichandran KS (2010) Clearance of apoptotic cells: implications in health and disease. J Cell Biol 189: 1059-1070. doi:10.1083/jcb.201004096. PubMed: 20584912. 20584912 10.1083/jcb.201004096 PMC2894449

[16] Gregory CD, Pound JD (2011) Cell death in the neighbourhood: direct microenvironmental effects of apoptosis in normal and neoplastic tissues. J Pathol 223: 177-194. PubMed: 21125674. 21125674 10.1002/path.2792

[17] Dini L (1998) Endothelial liver cell recognition of apoptotic peripheral blood lymphocytes. Biochem Soc Trans 26: 635-639. PubMed: 10047796. 10047796 10.1042/bst0260635

[18] Dini L, Autuori F, Lentini A, Oliverio S, Piacentini M (1992) The clearance of apoptotic cells in the liver is mediated by the asialoglycoprotein receptor. FEBS Lett 296: 174-178. doi:10.1016/0014-5793(92)80373-O. PubMed: 1370803. 1370803 10.1016/0014-5793(92)80373-o

[19] Dini L, Lentini A, Diez GD, Rocha M, Falasca L et al. (1995) Phagocytosis of apoptotic bodies by liver endothelial cells. J Cell Sci 108(3): 967-973. PubMed: 7622623. 7622623 10.1242/jcs.108.3.967

[20] Cao WM, Murao K, Imachi H, Hiramine C, Abe H et al. (2004) Phosphatidylserine receptor cooperates with high-density lipoprotein receptor in recognition of apoptotic cells by thymic nurse cells. J Mol Endocrinol 32: 497-505. doi:10.1677/jme.0.0320497. PubMed: 15072554. 15072554 10.1677/jme.0.0320497

[21] Monks J, Rosner D, Geske FJ, Lehman L, Hanson L et al. (2005) Epithelial cells as phagocytes: apoptotic epithelial cells are engulfed by mammary alveolar epithelial cells and repress inflammatory mediator release. Cell Death Differ 12: 107-114. doi:10.1038/sj.cdd.4401517. PubMed: 15647754. 15647754 10.1038/sj.cdd.4401517

[22] Monks J, Smith-Steinhart C, Kruk ER, Fadok VA, Henson PM (2008) Epithelial cells remove apoptotic epithelial cells during post-lactation involution of the mouse mammary gland. Biol Reprod 78: 586-594. doi:10.1095/biolreprod.107.065045. PubMed: 18057312. 18057312 10.1095/biolreprod.107.065045

[23] Erwig LP, Henson PM (2008) Clearance of apoptotic cells by phagocytes. Cell Death Differ 15: 243-250. doi:10.1038/sj.cdd.4402184. PubMed: 17571081. 17571081 10.1038/sj.cdd.4402184

[24] Medzhitov R, Janeway CA Jr. (1997) Innate immunity: the virtues of a nonclonal system of recognition. Cell 91: 295-298. doi:10.1016/S0092-8674(00)80412-2. PubMed: 9363937. 9363937 10.1016/s0092-8674(00)80412-2

[25] Franc NC, White K, Ezekowitz RA (1999) Phagocytosis and development: back to the future. Curr Opin Immunol 11: 47-52. doi:10.1016/S0952-7915(99)80009-0. PubMed: 10047544. 10047544 10.1016/s0952-7915(99)80009-0

[26] Gregory CD (2000) CD14-dependent clearance of apoptotic cells: relevance to the immune system. Curr Opin Immunol 12: 27-34. doi:10.1016/S0952-7915(99)00047-3. PubMed: 10679400. 10679400 10.1016/s0952-7915(99)00047-3

[27] Tennant I, Pound JD, Marr LA, Willems JJLP, Petrova S et al. (2012) Innate recognition of apoptotic cells: novel apoptotic cell-associated molecular patterns (ACAMPs) revealed by cross-reactivity of anti-LPS antibodies. Cell Death Differ (In Press). 10.1038/cdd.2012.165 PMC3619235 23392124

[28] Pugin J, Heumann ID, Tomasz A, Kravchenko VV, Akamatsu Y et al. (1994) CD14 is a pattern recognition receptor. Immunity 1: 509-516. doi:10.1016/1074-7613(94)90093-0. PubMed: 7534618. 7534618 10.1016/1074-7613(94)90093-0

[29] Devitt A, Moffatt OD, Raykundalia C, Capra JD, Simmons DL et al. (1998) Human CD14 mediates recognition and phagocytosis of apoptotic cells. Nature 392: 505-509. doi:10.1038/33169. PubMed: 9548256. 9548256 10.1038/33169

[30] Devitt A, Marshall LJ (2011) The innate immune system and the clearance of apoptotic cells. J Leukoc Biol 90: 447-457. doi:10.1189/jlb.0211095. PubMed: 21562053. 21562053 10.1189/jlb.0211095

[31] Wright SD, Ramos RA, Tobias PS, Ulevitch RJ, Mathison JC (1990) CD14, a receptor for complexes of lipopolysaccharide (LPS) and LPS binding protein. Science 249: 1431-1433. doi:10.1126/science.1698311. PubMed: 1698311. 1698311 10.1126/science.1698311

[32] Gregory CD, Devitt A (2003) Innate Immunity & Apoptosis: Cd14-dependent clearance of apoptotic cells. Apoptosis & Autoimmun: 111-132.

[33] Juan TS, Hailman E, Kelley MJ, Busse LA, Davy E et al. (1995) Identification of a lipopolysaccharide binding domain in CD14 between amino acids 57 and 64. J Biol Chem 270: 5219-5224. doi:10.1074/jbc.270.10.5219. PubMed: 7534291. 7534291 10.1074/jbc.270.10.5219

[34] Juan TS, Hailman E, Kelley MJ, Wright SD, Lichenstein HS (1995) Identification of a domain in soluble CD14 essential for lipopolysaccharide (LPS) signaling but not LPS binding. J Biol Chem 270: 17237-17242. doi:10.1074/jbc.270.29.17237. PubMed: 7542233. 7542233 10.1074/jbc.270.29.17237

[35] Viriyakosol S, Kirkland TN (1995) A region of human CD14 required for lipopolysaccharide binding. J Biol Chem 270: 361-368. doi:10.1074/jbc.270.1.361. PubMed: 7529231. 7529231 10.1074/jbc.270.1.361

[36] Stelter F, Bernheiden M, Menzel R, Jack RS, Witt S et al. (1997) Mutation of amino acids 39-44 of human CD14 abrogates binding of lipopolysaccharide and Escherichia coli. Eur J Biochem 243: 100-109. doi:10.1111/j.1432-1033.1997.00100.x. PubMed: 9030727. 9030727 10.1111/j.1432-1033.1997.00100.x

[37] Iwaki D, Nishitani C, Mitsuzawa H, Hyakushima N, Sano H et al. (2005) The CD14 region spanning amino acids 57-64 is critical for interaction with the extracellular Toll-like receptor 2 domain. Biochem Biophys Res Commun 328: 173-176. doi:10.1016/j.bbrc.2004.12.162. PubMed: 15670766. 15670766 10.1016/j.bbrc.2004.12.162

[38] Haziot A, Chen S, Ferrero E, Low MG, Silber R et al. (1988) The monocyte differentiation antigen, CD14, is anchored to the cell membrane by a phosphatidylinositol linkage. J Immunol 141: 547-552. PubMed: 3385210. 3385210

[39] Simmons DL, Tan S, Tenen DG, Nicholson-Weller A, Seed B (1989) Monocyte antigen CD14 is a phospholipid anchored membrane protein. Blood 73: 284-289. PubMed: 2462937. 2462937

[40] Jersmann HP (2005) Time to abandon dogma: CD14 is expressed by non-myeloid lineage cells. Immunol Cell Biol 83: 462-467. doi:10.1111/j.1440-1711.2005.01370.x. PubMed: 16174094. 16174094 10.1111/j.1440-1711.2005.01370.x

[41] Flora PK, Gregory CD (1994) Recognition of apoptotic cells by human macrophages: inhibition by a monocyte/macrophage-specific monoclonal antibody. Eur J Immunol 24: 2625-2632. doi:10.1002/eji.1830241109. PubMed: 7525298. 7525298 10.1002/eji.1830241109

[42] Prime SS, Nixon SV, Crane IJ, Stone A, Matthews JB et al. (1990) The behaviour of human oral squamous cell carcinoma in cell culture. J Pathol 160: 259-269. doi:10.1002/path.1711600313. PubMed: 1692339. 1692339 10.1002/path.1711600313

[43] Cunningham MD, Shapiro RA, Seachord C, Ratcliffe K, Cassiano L et al. (2000) CD14 employs hydrophilic regions to "capture" lipopolysaccharides. J Immunol 164: 3255-3263. PubMed: 10706718. 10706718 10.4049/jimmunol.164.6.3255

[44] Viriyakosol S, Kirkland TN (1996) The N-terminal half of membrane CD14 is a functional cellular lipopolysaccharide receptor. Infect Immun 64: 653-656. PubMed: 8550221. 8550221 10.1128/iai.64.2.653-656.1996 PMC173815

[45] Viriyakosol S, Mathison JC, Tobias PS, Kirkland TN (2000) Structure-function analysis of CD14 as a soluble receptor for lipopolysaccharide. J Biol Chem 275: 3144-3149. doi:10.1074/jbc.275.5.3144. PubMed: 10652298. 10652298 10.1074/jbc.275.5.3144

[46] Devitt A, Parker KG, Ogden CA, Oldreive C, Clay MF et al. (2004) Persistence of apoptotic cells without autoimmune disease or inflammation in CD14-/- mice. J Cell Biol 167: 1161-1170. doi:10.1083/jcb.200410057. PubMed: 15611337. 15611337 10.1083/jcb.200410057 PMC2172617

[47] Devitt A, Pierce S, Oldreive C, Shingler WH, Gregory CD (2003) CD14-dependent clearance of apoptotic cells by human macrophages: the role of phosphatidylserine. Cell Death Differ 10: 371-382. doi:10.1038/sj.cdd.4401168. PubMed: 12700637. 12700637 10.1038/sj.cdd.4401168

[48] Erb-Downward JR, Thompson DL, Han MK, Freeman CM, McCloskey L et al. (2011) Analysis of the lung microbiome in the "healthy" smoker and in COPD. PLOS ONE 6: e16384. doi:10.1371/journal.pone.0016384. PubMed: 21364979. 21364979 10.1371/journal.pone.0016384 PMC3043049

[49] Shiratsuchi A, Watanabe I, Takeuchi O, Akira S, Nakanishi Y (2004) Inhibitory effect of Toll-like receptor 4 on fusion between phagosomes and endosomes/lysosomes in macrophages. J Immunol 172: 2039-2047. PubMed: 14764668. 14764668 10.4049/jimmunol.172.4.2039

[50] Poon IK, Hulett MD, Parish CR (2010) Molecular mechanisms of late apoptotic/necrotic cell clearance. Cell Death Differ 17: 381-397. doi:10.1038/cdd.2009.195. PubMed: 20019744. 20019744 10.1038/cdd.2009.195

[51] Triantafilou M, Miyake K, Golenbock DT, Triantafilou K (2002) Mediators of innate immune recognition of bacteria concentrate in lipid rafts and facilitate lipopolysaccharide-induced cell activation. J Cell Sci 115: 2603-2611. PubMed: 12045230. 12045230 10.1242/jcs.115.12.2603

[52] Hyakushima N, Mitsuzawa H, Nishitani C, Sano H, Kuronuma K et al. (2004) Interaction of soluble form of recombinant extracellular TLR4 domain with MD-2 enables lipopolysaccharide binding and attenuates TLR4-mediated signaling. J Immunol 173: 6949-6954. PubMed: 15557191. 15557191 10.4049/jimmunol.173.11.6949

[53] Huyton T, Rossjohn J, Wilce M (2007) Toll-like receptors: structural pieces of a curve-shaped puzzle. Immunol Cell Biol 85: 406-410. doi:10.1038/sj.icb.7100089. PubMed: 17607319. 17607319 10.1038/sj.icb.7100089

[54] Kim JI, Lee CJ, Jin MS, Lee CH, Paik SG et al. (2005) Crystal structure of CD14 and its implications for lipopolysaccharide signaling. J Biol Chem 280: 11347-11351. doi:10.1074/jbc.M414607200. PubMed: 15644310. 15644310 10.1074/jbc.M414607200

[55] Shapiro RA, Cunningham MD, Ratcliffe K, Seachord C, Blake J et al. (1997) Identification of CD14 residues involved in specific lipopolysaccharide recognition. Infect Immun 65: 293-297. PubMed: 8975926. 8975926 10.1128/iai.65.1.293-297.1997 PMC174590

[56] Stelter F, Loppnow H, Menzel R, Grunwald U, Bernheiden M et al. (1999) Differential impact of substitution of amino acids 9-13 and 91-101 of human CD14 on soluble CD14-dependent activation of cells by lipopolysaccharide. J Immunol 163: 6035-6044. PubMed: 10570291. 10570291

[57] Moffatt O, Ferguson E, Devitt A, Flora P, Simmons DL et al. (1996) Involvement of ICAM-3 in the interaction of apoptotic cells with macrophages. Immunology 89: R176-R176.

[58] Henriksen PA, Devitt A, Kotelevtsev Y, Sallenave JM (2004) Gene delivery of the elastase inhibitor elafin protects macrophages from neutrophil elastase-mediated impairment of apoptotic cell recognition. FEBS Lett 574: 80-84. doi:10.1016/j.febslet.2004.08.008. PubMed: 15358543. 15358543 10.1016/j.febslet.2004.08.008

[59] Regueiro V, Campos MA, Morey P, Sauleda J, Agustí AG et al. (2009) Lipopolysaccharide-binding protein and CD14 are increased in the bronchoalveolar lavage fluid of smokers. Eur Respir J 33: 273-281. PubMed: 19010986. 19010986 10.1183/09031936.00087708

[60] Julius P, Grosse-Thie C, Kuepper M, Bratke K, Virchow JC (2010) sCD14 in bronchoalveolar lavage 18: 42 and 162 hours after segmental allergen provocation. Scand J Immunol 71: 304-311 10.1111/j.1365-3083.2010.02375.x 20384875

[61] Henson PM, Vandivier RW, Douglas IS (2006) Cell death, remodeling, and repair in chronic obstructive pulmonary disease? Proc Am Thorac Soc 3: 713-717. doi:10.1513/pats.200605-104SF. PubMed: 17065379. 17065379 10.1513/pats.200605-104SF PMC2647658

