# Supplementary figures and images for: The N-Terminus of CD14 Acts to Bind Apoptotic Cells and Confers Rapid-Tethering Capabilities on Non-Myeloid Cells
Source: PLoS One. 2013 Jul 30;8(7):e70691. doi: 10.1371/journal.pone.0070691 (PMC3728300; doi:10.1371/journal.pone.0070691)

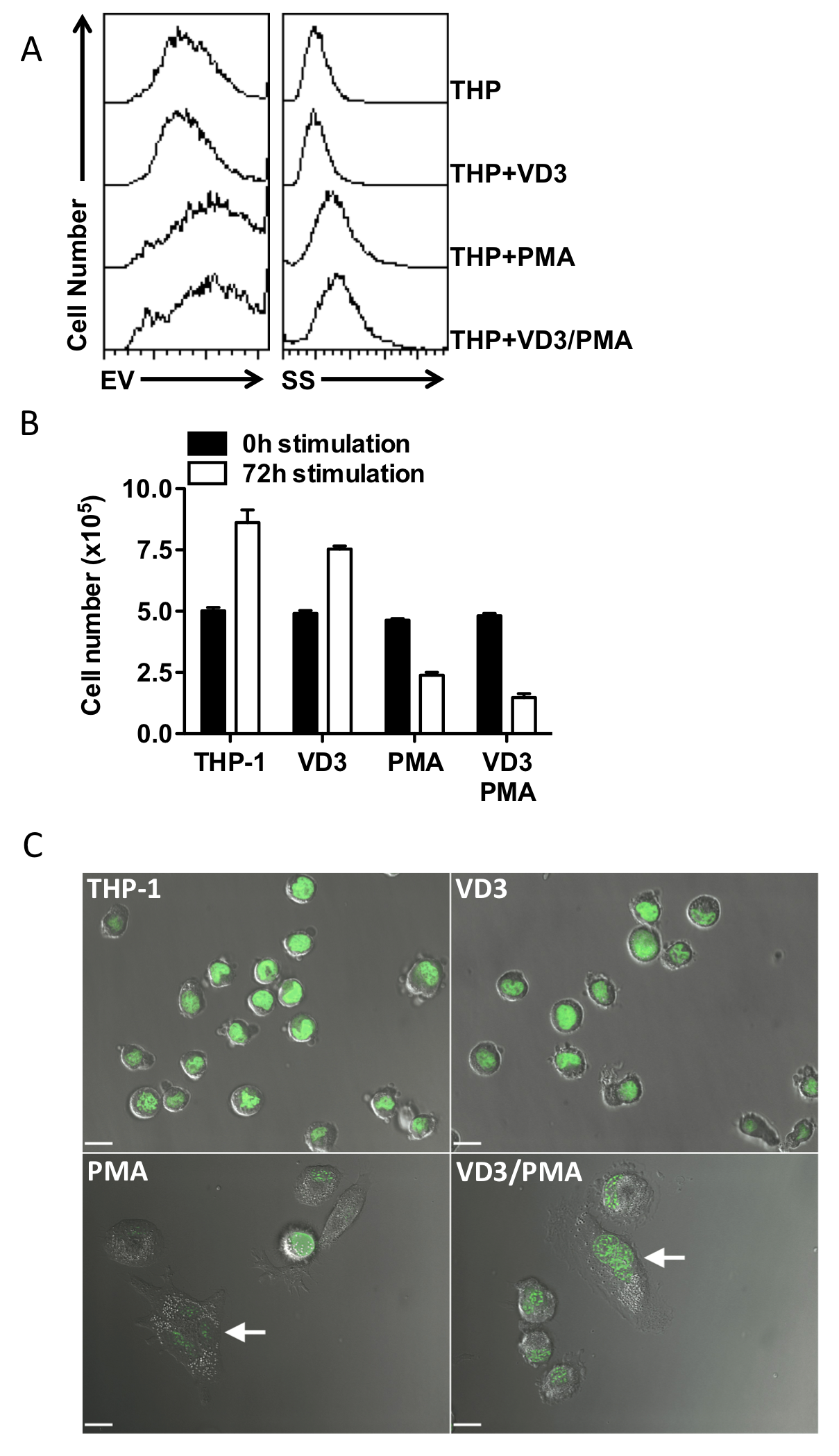

Supplement: Figure S1 — (A) THP-1 monocytes (THP-1) cells were stimulated to differentiate in the presence of dihydroxyvitamin D3 (VD3), phorbol ester (PMA) or both (VD3/PMA) for 48 hours. Resultant cells were detached into 5mM EDTA in PBS by incubation at 37°C for 15 min prior to flow cytometric analysis of cell volume. Data shown are representative of the electronic volume frequency histograms for the resultant cell populations. (B) THP-1 cells were seeded at a density of 5x10^5^ cells per well prior to mock treatment (THP-1) or treatment with 100nM dihydroxyvitamin D3 (VD3), 250nM phorbol ester (PMA) or VD3/PMA. Following 72 hours, cell numbers were assessed using the cell count function of the Quanta SC flow cytometer. Data shown are the mean ± SE of cell counts from three independent experiments. (C) THP-1 monocytes (THP-1) cells were stimulated to differentiate in the presence of dihydroxyvitamin D3 (VD3), phorbol ester (PMA) or both (VD3/PMA) for 48 hours in 4 well chamber slides. Cell nuclei were stained with acridine orange. Representative DIC morphology images overlaid with fluorescence nuclear morphology images of THP-1 cells or the resultant differentiated cell are shown. Multinucleate cells, suggestive of cell fusion, are shown (arrows). Scale bar = 16µm. (TIF) [file pone.0070691.s001.tif]

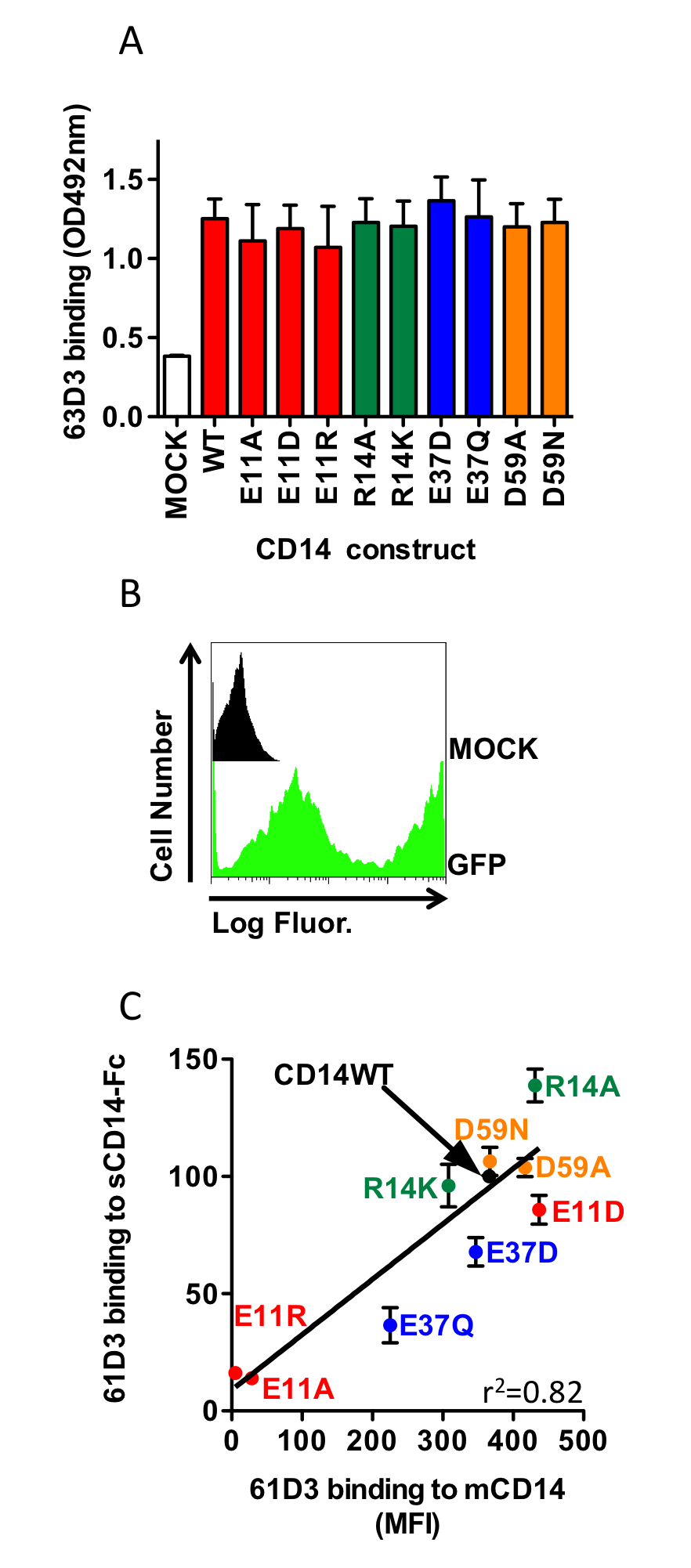

Supplement: Figure S2 — (A) Monoclonal Ab 63D3 was tested for reactivity against wild-type CD14 and a panel of point mutants. Anti-human Fc immobilised soluble CD14-Fc fusion proteins were probed by ELISA with mAb 63D3 and binding detected with anti-mouse-HRP prior to developing with OPD substrate and reading OD492nm. Data shown are mean ± SE of three independent experiments. Statistical analyses indicate no significant difference in response to any of the CD14 constructs (ANOVA with Dunnett’s post-test). (B) HeLa cells were transfected with pcDNA3/GFP. The fluorescence frequency histogram shown reveals the representative bi-modal expression pattern noted in all our HeLa cell studies. (C) Regression analysis of 61D3 mapping studies on soluble CD14 constructs (WT and point mutants) and HeLa cell membrane expressed constructs. Binding of 61D3 to sCD14 is plotted against the mean fluorescence intensity of 61D3 stained HeLa transfectants (all data from Figure 4). This analysis reveals a strong correlation between 61D3 mapping on soluble and membrane CD14 with a correlation coefficient (r) = 0.905. (TIF) [file pone.0070691.s002.tif]

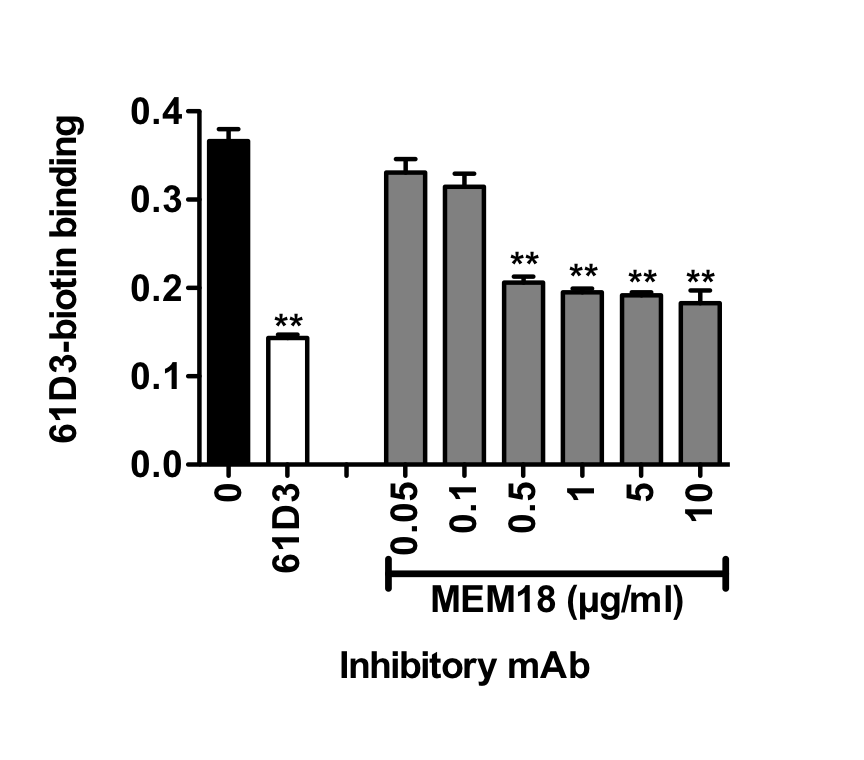

Supplement: Figure S3 — Anti-human Fc immobilised soluble WT CD14-Fc fusion protein was probed by ELISA with mAb 61D3-biotin and binding of the biotinylated mAb detected with streptavidin-HRP prior to developing with OPD substrate and reading OD492nm. The ability of unlabelled 61D3 (red bar) or unlabelled MEM18 (blue bars, used at indicated concentrations) to block binding of biotinylated 61D3 was assessed. Data shown are mean ± SE of three independent experiments. Statistical analyses used ANOVA with Dunnett’s post-test to detect significant of differences compared to 61D3-biotin alone (black bar). (TIF) [file pone.0070691.s003.tif]

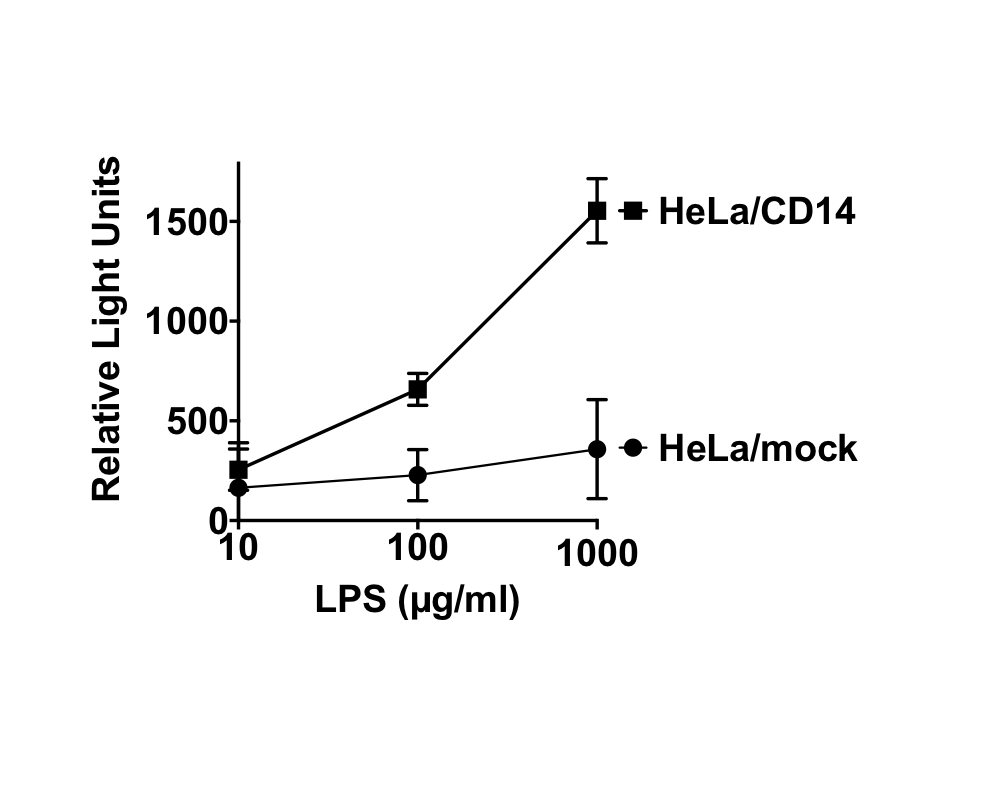

Supplement: Figure S4 — HeLa cells were transfected with both the luciferase NFκB reporter plasmid and a CD14WT expression plasmid or ICAM-3 expression plasmid as a control using TransIT LT-1. Expression was allowed to proceed for 24 hours prior to further analyses. Cells were treated with the indicated concentrations of LPS for 5h prior to assessing NFκB-mediated transcriptional activity with One-Glo Luciferase assay system. Relative light units were quantified using a microplate luminometer. The data shown is mean ± SE of three independent experiments. Statistical analyses used ANOVA with Tukey post-test. *P<0.05. (TIF) [file pone.0070691.s004.tif]
